# A conforming auxiliary space preconditioner for the mass conserving stress‐yielding method

**DOI:** 10.1002/nla.2503

**Published:** 2023-05-07

**Authors:** Lukas Kogler, Philip L. Lederer, Joachim Schöberl

**Affiliations:** ^1^ Institute for Analysis and Scientific Computing TU Wien Vienna Austria

**Keywords:** auxiliary space preconditioner, exact divergence‐free velocity, high order robustness, iterative solver, Stokes equations

## Abstract

We are studying the efficient solution of the system of linear equations stemming from the mass conserving stress‐yielding (MCS) discretization of the Stokes equations. We perform static condensation to arrive at a system for the pressure and velocity unknowns. An auxiliary space preconditioner for the positive definite velocity block makes use of efficient and scalable solvers for conforming Finite Element spaces of low order and is analyzed with emphasis placed on robustness in the polynomial degree of the discretization. Numerical experiments demonstrate the potential of this approach and the efficiency of the implementation.

## 1 INTRODUCTION

Let $\Omega \subset {\mathbb{R}}^{d}$ be a bounded domain with d=2 or 3 with Lipschitz boundary Γ:=∂Ω. Let u and p be the velocity and the pressure, respectively. Given an external body force $f:\Omega \to {\mathbb{R}}^{d}$ and the double of kinematic viscosity denoted by ν, the velocity‐pressure formulation of the Stokes system is given by

(1a) $$\begin{array}{ll} -\;\text{div}(\nu \varepsilon (u))+\nabla p & =f\;\text{in}\;\Omega ,\; \end{array}$$

(1b) $$\begin{array}{ll} \text{div}(u) & =0\;\text{in}\;\Omega , \end{array}$$

where $\varepsilon (u)=\frac{1}{2}(\nabla u+{\left(\nabla u\right)}^{T})$. By introducing additional matrix valued variables σ:=−νε(u) for the stress and $\omega :=\frac{1}{2}(\nabla u-{\left(\nabla u\right)}^{T})$, these equations can be restated as

(2a) $$\begin{array}{ll} -\;\nu^{-1}\text{dev}(\sigma )-\nabla u+\omega & =0\;\text{in}\;\Omega ,\; \end{array}$$

(2b) $$\begin{array}{ll} \text{div}(\sigma )+\nabla p & =f\;\text{in}\;\Omega ,\; \end{array}$$

(2c) $$\begin{array}{ll} \sigma -\sigma^{T} & =0\;\text{in}\;\Omega , \end{array}$$

(2d) $$\begin{array}{ll} \;\text{div}(u) & =0\;\text{in}\;\Omega , \end{array}$$

where dev(σ) denotes the deviatoric part (see Section 2), and (2a) is motivated by the fact that for the solution of (1) we have σ=−νε(u)=−νdev(ε(u))=dev(σ). The introduction of ω as a Lagrange multiplier enables the derivation of discrete methods that enforce the symmetry constraint (2c) weakly, see also References 1, 2, 3. As boundary conditions, we consider Dirichlet ones for the velocity u, homogenous purely for clarity of the presentation, and two kinds of outlet conditions,

(2e) $$\begin{array}{ll} \;u & =0\;\text{on}\;\Gamma_{D}, \end{array}$$

(2f) $$\begin{array}{ll} \;(\sigma +pI)n & =0\;\text{on}\;\Gamma_{N}, \end{array}$$

(2g) $$\begin{array}{ll} ((\sigma +pI)n)\cdot n=u_{t} & =0\;\text{on}\;\Gamma_{Ñ}, \end{array}$$

where I is the d×d identity matrix and $u_{t}$ is the tangential part of u. We assume that both $\Gamma_{D}$ and at least one of $\Gamma_{N}$ or $\Gamma_{Ñ}$ have positive measure. As usual, when $\Gamma_{N}=\Gamma_{Ñ}=\emptyset$, an additional condition must be imposed on the pressure to make it unique.

In recent years, divergence‐free and pressure‐robust Finite Element discretizations, that is those whose solutions fulfill (2d) strongly, and allow for pressure‐independent a‐priori error estimates respectively, have been of great interest.^4^

For the velocity‐pressure formulation (1), one class of such methods are certain Hybrid Discontinuous Galerkin (HDG) methods that take the velocity in H(div,Ω) and the pressure in $L^{2}(\Omega )$, that is, they only build normal continuity into the Finite Element space while the tangential continuity of the solution is enforced via Lagrange parameters. To make the resulting system for the velocity positive definite, a consistent stabilization term has to be added, often involving either a parameter that has to be sufficiently large or a lifting of the jump, see References 5, 6.

In References 7, 8, the authors presented a novel variational formulation for the Stokes equations that still takes the velocity in H(div,Ω) and pressure in $L^{2}(\Omega )$, retaining the property of yielding exactly divergence‐free and pressure‐robust solutions, but is based on (2) instead of (1). This mass conserving stress‐yielding (MCS) method features a normal‐tangential continuous stress space and requires no stabilizing term. It was already remarked in the original work^7^ that static condensation can be performed to eliminate certain σ degrees of freedom (dofs). Later, in Reference 9, the normal‐tangential continuity of σ was broken and instead an additional Lagrange parameter û was introduced. This technique makes it possible to eliminate σ altogether and to reduce the problem to one for the velocity in H(div,Ω), the pressure in $L^{2}(\Omega )$, and the newly introduced û which approximates the tangential velocity trace on the mesh facets. The velocity unknowns u,û take the place of σ as primal variables in the condensed saddle point system, with the pressure remaining the Lagrange parameter enforcing (2d). The condensed system involves the same variables, and has the same structure as the above HDG methods, but does *not require a stabilization term*. The first contribution of this work is a characterization of the velocity block of the condensed system based on its relation to the velocity block resulting from an HDG method with optimal stabilization. In particular, we prove that the velocity block, as previously claimed in Reference 9 for that of a related low order MCS method, is indeed positive definite.

We then move on to the question of how to efficiently solve the condensed system and consider preconditioned Krylov space methods. Preconditioning techniques for saddle point systems based on separate preconditioners for the primal (velocity) and Lagrange (pressure) unknowns are a well studied subject, see Reference 10, and the pressure Schur complement is easily preconditioned, see Reference 11. Therefore, our focus is on identifying and analyzing suitable preconditioners for the condensed velocity block.

The literature on preconditioners for conforming methods is vast and includes, among others, domain decomposition, see Reference 12, as well as Geometric, see Reference 13, and Algebraic, see Reference 14, Multigrid methods and an even somewhat comprehensive review would be beyond the scope of this work. We will take as given that efficient and scalable solvers for conforming methods exist and are available.

Preconditioners for HDG methods are not quite as well studied in the literature, one recurring theme is the attempt to reuse conforming preconditioners for these non‐conforming spaces. For example, a non‐nested Multigrid method with conforming coarse grid spaces was studied in Reference 15, and auxiliary space preconditioners (ASP, see Reference 16) that also feature a conforming sub‐space were considered in Reference 17.

The idea at the heart of both approaches is to decompose functions in the non‐conforming space into a conforming component plus a (small) remainder and to address them separately with some pre‐existing conforming preconditioner and a simple, computationally inexpensive method such as (Block‐)Jacobi, respectively.

The principal focus in this work is on the introduction and analysis of ASPs for the MCS method. The main improvement over the theory in Reference 17 is that the analysis of the velocity preconditioners extends techniques from Reference 18 and is explicit in the polynomial degree of the discretization. In particular, the main result, Theorem 3, states that the condition number of a particular ASP is bounded by $\gamma \cdot {\left(\log(k)\right)}^{3}$, where k is the polynomial degree of the discretization and γ is a constant stemming from the relation between condensed MCS and HDG norms.

We close out the discussion with numerical experiments that demonstrate the robustness and scalability of the proposed preconditioners. It is a testament to the elegance and simplicity of the ASP method that we were able to scale the computations to a relatively large scale by leveraging existing, scalable and highly Performant software.

**Outline**

We gather notation used throughout this work in Section 2 and introduce various Finite Element spaces and norms in Section 3 which also contains some useful technical results. Section 4 reviews the MCS method itself and contains a thorough discussion of static condensation as well as results on the obtained condensed systems. Approaches for preconditioning saddle point matrices with separate preconditioners for the primal unknowns and Lagrange multipliers as well as the method of auxiliary space preconditioning are recalled in Section 5. The main results can be found in Section 6, where different variations of ASPs for the velocity block of the Stokes system are discussed. In Section 7, we sketch the treatment of the lowest order case which is not covered by the theory developed in previous sections. Finally, numerical experiments are performed in Section 8.

## 2 NOTATION

With 𝕄 denoting the vector space of real d×d matrices, we define the subsets of skew‐symmetric and skew‐symmetric trace‐free matrices by

$$\begin{array}{ll} 𝕂=\{\tau \in 𝕄:\tau +\tau^{T}=0\}\;\text{and}\;𝔻=\{\tau \in 𝕄:\tau :I=0\}, & \end{array}$$

where ${\left(\cdot \right)}^{T}$ denotes the transpose and I∈𝕄 the identity matrix. To differentiate between scalar‐, vector‐ and matrix‐valued functions on some subset D⊆Ω we include the range in the notation for the latter two while we omit it for the former one, i.e. where $L^{2}(D,\mathbb{R})=L^{2}(D)$ denotes the space of square integrable ℝ‐valued scalar functions, the spaces $L^{2}(D,{\mathbb{R}}^{d})$ and $L^{2}(D,𝕄)$ denote the analogous vector‐ and matrix‐valued spaces. Similarly, ${\mathbb{P}}^{k}(D,\mathbb{R})={\mathbb{P}}^{k}(D)$, and so forth, denote the set of scalar‐, vector‐ or matrix‐valued polynomials up to degree k on D. We use the notation ${\left(\cdot ,\cdot \right)}_{D}$ for the $L^{2}$‐inner product on D and set $\|\cdot {\|}_{D}^{2}={\left(\cdot ,\cdot \right)}_{D}$. The $L^{2}$‐orthogonal projection onto ${\mathbb{P}}^{k}(D,\cdot )$ (the range should be clear from context) is denoted by $\Pi_{D}^{k}$ and we will occasionally omit the subscript. Similarly, the $L^{2}$‐orthogonal projector onto the (restrictions to D of) the rigid body modes $R_{D}:=\{u(x)=a+b\times x:a,b\in {\mathbb{R}}^{d}\}$ is written as $\Pi_{D}^{R}$.

In the following, let ϕ, Φ, and Ψ be smooth scalar‐, vector‐, and matrix‐valued functions, respectively. The operator ∇ is to be understood from context as resulting in either in a vector whose components are $\partial_{i}\phi :=\partial \phi /\partial x_{i}$ or a matrix with components $\left(\partial_{i}\Phi_{j}\right)$. For vector‐valued functions in three dimensions the operator curl is defined as curlΦ:=∇×Φ and in two we understand it to refer to the scalar‐valued $\text{curl}\;\phi :=-\partial_{2}\phi_{1}+\partial_{1}\phi_{2}$. The divergence operator div is understood as $\text{div}\Phi :=\sum_{j=1}^{d}\partial_{j}\Phi_{j}$ for vectors and is applied row‐wise to matrices, that is, ${\left(\text{div}\Psi \right)}_{i}:=\sum_{j=1}^{d}\partial_{j}\Psi_{ij}$. Besides the well known trace operator $\text{tr}(\Psi ):=\sum_{j=1}^{d}\Psi_{ii}$ and the deviatoric part $\text{dev}(\Psi ):=\Psi -\frac{1}{d}\text{tr}(\Psi )I$ we further introduce the operator $\kappa :{\mathbb{R}}^{d(d-1)/2}\to 𝕂$ by

$$\begin{array}{ll} \kappa (\phi ):=\frac{1}{2}\left(\begin{array}{cc} 0 & -\phi \\ \phi & 0 \end{array}\right)\;\text{if}\;d=2,\;\kappa (\Phi ):=\frac{1}{2}\left(\begin{array}{ccc} 0 & -\Phi_{3} & \Phi_{2}\\ \Phi_{3} & 0 & -\Phi_{1}\\ -\;\Phi_{2} & \Phi_{1} & 0 \end{array}\right)\;\text{if}\;d=3. & \end{array}$$

Based on these differential operators, we use standard notation for the Sobolev spaces $H^{m}(\Omega ,\mathbb{R})=H^{m}(\Omega ),H(\text{div},\Omega )$ and H(curl,Ω) with m≥0. Further, for some $\Gamma_{*}\subseteq \partial \Omega$, a subscript “$0,\Gamma_{*}$” indicates that the corresponding natural traces vanish on $\Gamma_{*}$, and we use only the zero subscript if $\Gamma_{*}=\partial \Omega$.

We denote by ${𝒯}_{h}$ a quasi‐uniform and shape regular triangulation of the domain Ω into simplices. Let h denote the maximum of the diameters of all elements in ${𝒯}_{h}$. The set of element interfaces and boundaries, or facets, is denoted by ${ℱ}_{h}$ and the set of facets of a particular element $T\in {𝒯}_{h}$ is ${ℱ}_{T}:=\{F\in {ℱ}_{h}:F\subseteq \partial T\}$. By an abuse of notation, we shall also use ${ℱ}_{h}$ to denote the domain formed by union of all $F\in {ℱ}_{h}$. We assume that the mesh resolves the domain boundary parts in the sense that $\forall F\in {ℱ}_{h}$ with $F\subseteq \partial \Omega \;\exists !\Gamma_{*}\in \{\Gamma_{D},\Gamma_{N},\Gamma_{Ñ}\}$ such that $F\subseteq \Gamma_{*}$. This splits ${ℱ}_{h}$ into boundary facets ${ℱ}_{h}^{D}:=\{F\in {ℱ}_{h}:F\subseteq \Gamma_{D}\}$, ${ℱ}_{h}^{N}:=\{F\in {ℱ}_{h}:F\subseteq \Gamma_{N}\}$, and ${ℱ}_{h}^{Ñ}:=\{F\in {ℱ}_{h}:F\subseteq \Gamma_{Ñ}\}$, and interior facets ${ℱ}_{h}^{0}:={ℱ}_{h}\setminus ({ℱ}_{h}^{D}\cup {ℱ}_{h}^{N}\cup {ℱ}_{h}^{Ñ})$. According to this mesh we also introduce the “broken” spaces

$$\begin{array}{ll} H^{m}({𝒯}_{h},\cdot ):=\underset{T\in {𝒯}_{h}}{\prod }H^{m}(T,\cdot ),\;{\mathbb{P}}^{k}({𝒯}_{h},\cdot ):=\underset{T\in {𝒯}_{h}}{\prod }{\mathbb{P}}^{k}(T,\cdot ),\;{\mathbb{P}}^{k}({ℱ}_{h},\cdot ):=\underset{F\in {ℱ}_{h}}{\prod }{\mathbb{P}}^{k}(F,\cdot ), & \end{array}$$

where, as before, we include the range explicitly, for example, as in ${\mathbb{P}}^{k}({𝒯}_{h},{\mathbb{R}}^{d})$. On each $F\in {ℱ}_{h}$ we denote by ⟦·⟧ and {{·}} the standard jump and mean value operators and take them to be the identity on boundary facets. On each element boundary and each facet $F\in {ℱ}_{h}$ we denote by n the outward unit normal vector. The scalar normal and vector‐valued tangential traces of a sufficiently smooth function v are given by $v_{n}:=v\cdot n$ and $v_{t}:=v-v_{n}n$. Similarly, the normal‐normal and normal‐tangential traces of a smooth matrix‐valued function Ψ are $\Psi_{nn}:=\Psi :(n⊗n)=n^{T}\Psi n$ and $\Psi_{nt}=\Psi n-\Psi_{nn}n$.

We write functions in general Sobolev spaces as u,û,ω, and so forth, discrete functions with a subscript h as $u_{h},{û}_{h},\omega_{h}$, and so forth, and their via Galerkin isomorphism identified coefficient vectors w.r.t to some given Finite Element basis as $u,\hat{u},\omega$, and so forth. For readability of the presentation we make no difference between row and column vectors and, for example, write (u,û) for the coefficient vector of $\left(u_{h},{û}_{h}\right)$ which should strictly speaking be the column vector ${\left(u^{T},{û}^{T}\right)}^{T}$. Similarly, operators are capital letters A,B, and so forth, their discrete counterparts $A_{h},B_{h}$, and so forth, and the corresponding Finite Element matrices A,B, etc. Occasionally, when it is useful to emphasize the Galerkin isomorphism we use ${∼}_{G}$, for example, $u_{h}{∼}_{G}u$ or $A_{h}{∼}_{G}A$.

Finally, throughout this work we write A≲B when there exists a constant c>0 *independent of the mesh size* h *and the viscosity* ν such that cA≤B and A∼B⇔A≲B∧B≲A. For example, due to quasi‐uniformity we have $h∼\text{diam}(T)\;\forall T\in {𝒯}_{h}$. For two elliptic operators A,B (or symmetric and positive definite matrices A,B) we take A≲B to mean that the maximum eigenvalue of the generalized eigenvalue problem Ax=λBx is bounded by a constant C similarly independent of h and ν. Note that in inequalities related to discrete functions or operators, unless explicitly stated otherwise, these constants can depend on the polynomial degree. Henceforth we assume that ν is a constant.

## 3 FINITE ELEMENTS AND NORM EQUIVALENCES

Reminding our self that the lowest order case is addressed separately in Section 7, we define the following approximation spaces for k≥2:

(3) $$\begin{array}{ll} V_{h} & :=\{u_{h}\in {\text{BDM}}^{k}({𝒯}_{h}):{\left(u_{h}\right)}_{n}=0\;\text{on}\;\Gamma_{D}\},\; \end{array}$$

(4) $$\begin{array}{ll} {\hat{V}}_{h} & :=\{{û}_{h}\in {\mathbb{P}}^{k-1}({ℱ}_{h},{\mathbb{R}}^{d}):{\left({û}_{h}\right)}_{n}=0\;\forall F\in {ℱ}_{h}\;\text{and}\;{û}_{h}=0\;\forall F\subset \Gamma_{D}\cup \Gamma_{Ñ}\}, \end{array}$$

(5) $$\begin{array}{ll} W_{h} & :={\mathbb{P}}^{k-1}({𝒯}_{h},𝕂),\; \end{array}$$

(6) $$\begin{array}{ll} \sum_{h} & :=\{\tau_{h}\in {\mathbb{P}}^{k}({𝒯}_{h},𝔻):{\left(\tau_{h}\right)}_{nt}\in {\mathbb{P}}^{k-1}(F,{\mathbb{R}}^{d})\;\forall F\in {ℱ}_{h}\},\; \end{array}$$

(7) $$\begin{array}{ll} Q_{h} & :={\mathbb{P}}^{k-1}({𝒯}_{h},\mathbb{R}),\; \end{array}$$

(8) $$\begin{array}{ll} {\bar{V}}_{h} & :=\{u_{h}\in {\mathbb{P}}^{1}({𝒯}_{h},{\mathbb{R}}^{d})\cap H^{1}(\Omega ,{\mathbb{R}}^{d}):u_{h}=0\;\text{on}\;\Gamma_{D}\}.\; \end{array}$$

See Reference 19 for a detailed discussion of the H(div)‐conforming Brezzi‐Douglas‐Marini (BDM) space appearing in the definition of $V_{h}$. Note that, restricted to a single element T, in addition to ${\mathbb{P}}^{k-1}(T,𝔻)$, the stress space $\sum_{h}$ also includes functions in ${\mathbb{P}}^{k}(T,𝔻)$ with vanishing normal tangential trace (“nt‐bubbles”). We further define the space of divergence free velocities $V_{h}^{0}:=\{v_{h}\in V_{h}:\text{div}(v_{h})=0\}$ and the product spaces ${𝒱}_{h}:=V_{h}\times {\hat{V}}_{h}$, $U_{h}:=V_{h}\times {\hat{V}}_{h}\times W_{h}$ and $U_{h}^{0}:=V_{h}^{0}\times {\hat{V}}_{h}\times W_{h}$. Following Reference 20, for $T\in {𝒯}_{h}$, $F\in {ℱ}_{T}$ and $u\in {\mathbb{P}}^{k}(F,{\mathbb{R}}^{d})$ we write

(9) $$\begin{array}{ll} \|u{\|}_{j,F,T}^{2}:=\underset{\sigma \in {\mathbb{P}}^{k}(T,{\mathbb{R}}^{d})}{\sup}\frac{{\left(u,\sigma \right)}_{F}^{2}}{\|\sigma {\|}_{T}^{2}}∼h^{-1}\overset{k}{\underset{j=0}{\sum }}k(k-j+1)\|(\Pi_{F}^{j}-\Pi_{F}^{j-1})u{\|}_{F}^{2}, & \end{array}$$

where $\Pi_{F}^{-1}:=0$ and the equivalence was shown in [Theorem 2].^20^ Where it is clear from the context which volume element T is meant, we omit it from the subscript and simply write $\|\cdot {\|}_{j,F}$. We define Hybrid Discontinuous Galerkin (HDG) norms on ${𝒱}_{h}$ and $U_{h}$ by

(10) $$\begin{array}{ll} \|(u_{h},{û}_{h}){\|}_{\varepsilon ,h}^{2} & :=\underset{T\in {𝒯}_{h}}{\sum }(\|\varepsilon (u_{h}){\|}_{T}^{2}+\underset{F\in {ℱ}_{T}}{\sum }\|\Pi^{k-1}{\left(u_{h}-{û}_{h}\right)}_{t}{\|}_{j,F}^{2}),\; \end{array}$$

(11) $$\begin{array}{ll} \|(u_{h},{û}_{h},\omega_{h}){\|}_{U_{h}}^{2} & :=\underset{T\in {𝒯}_{h}}{\sum }\|\varepsilon (u_{h}){\|}_{T}^{2}+\|\kappa (\text{curl}(u_{h}))-\omega_{h}{\|}_{T}^{2}+h^{-1}\|\Pi^{k-1}{\left(u_{h}-{û}_{h}\right)}_{t}{\|}_{\partial T}^{2}, \end{array}$$

(12) $$\begin{array}{ll} |(u_{h},{û}_{h},\omega_{h}){|}_{U_{h},*}^{2} & :=\underset{T\in {𝒯}_{h}}{\sum }\|\text{dev}(\nabla u-\omega_{h}){\|}_{T}^{2}+h^{-1}\|\Pi^{k-1}{\left(u_{h}-{û}_{h}\right)}_{t}{\|}_{\partial T}^{2}.\; \end{array}$$

In (10), the terms for $F\in \Gamma_{Ñ}$, where ${û}_{h}=0$, weakly enforce $u_{t}=0$ from (2g). There holds the equivalence (see Reference 9)

(13) $$\|(u_{h},{û}_{h},\omega_{h}){\|}_{U_{h}}^{2}∼|(u_{h},{û}_{h},\omega_{h}){|}_{U_{h},*}^{2}+d^{-1}\|\text{div}(u_{h}){\|}_{0}^{2}\;\forall (u_{h},{û}_{h},\omega_{h})\in U_{h}.$$

### 3.1 Technical results

For readability, the technical details of this Section are moved to the Appendix.

#### 3.1.1 Interpolation operators

A well known interpolation operator ${ℐ}_{f}:H^{2}({𝒯}_{h},{\mathbb{R}}^{d})\to {\bar{V}}_{h}^{f}:={\mathbb{P}}^{1}({𝒯}_{h},{\mathbb{R}}^{d})\cap H^{1}(\Omega ,{\mathbb{R}}^{d})$ is defined by

(14) $$\begin{array}{ll} ({ℐ}_{f}(u))(p)=\frac{1}{\left|\chi_{p}\right|}\underset{T\in \chi_{p}}{\sum }u_{|T}(p)\;\forall p\in 𝒱, & \end{array}$$

where $\chi_{p}$ is the set of all elements that share the vertex p, and $\left|\chi_{p}\right|$ is the number of such elements. Bounds for the approximation error of ${ℐ}_{f}$ in $H^{1}$‐like norms are very standard and well known, and with a Korn inequality for broken $H^{1}$ spaces like

(15) $$\begin{array}{ll} \underset{T\in {𝒯}_{h}}{\sum }\|\nabla u{\|}_{T}^{2}\leq C_{K}\underset{T\in {𝒯}_{h}}{\sum }\|\varepsilon (u){\|}_{T}^{2}+\underset{F\in {ℱ}_{h}^{0}\cup {ℱ}_{h}^{D}}{\sum }\|\Pi_{F}^{R}⟦u⟧{\|}_{F}^{2}\;\forall u\in H^{1}({𝒯}_{h},{\mathbb{R}}^{d}), & \end{array}$$

derived in Reference 21, it can easily be bounded by an $\|\cdot {\|}_{\varepsilon ,h}$ like one. However, as the kernel of ε is controlled only by the ${ℱ}_{h}^{D}$ terms, $C_{k}$ can degenerate depending on the shape of Ω and $\Gamma_{D}$. As it would otherwise later on enter into condition number estimates, the following Lemma 1 bounds the approximation error of ${ℐ}_{f}$ independent of $C_{K}$.

Lemma 1 There holds

(16) $$\begin{array}{ll} \underset{T\in {𝒯}_{h}}{\sum }h^{-2}\|u & -{ℐ}_{f}u{\|}_{T}^{2}+\|\nabla (u-{ℐ}_{f}u){\|}_{T}^{2}≲\underset{T\in {𝒯}_{h}}{\sum }\|\varepsilon (u){\|}_{T}^{2}+\underset{F\in {ℱ}_{h}^{0}}{\sum }h^{-1}\|\Pi_{F}^{R}⟦u⟧{\|}_{F}^{2}\;\forall u\in H^{2}({𝒯}_{h},{\mathbb{R}}^{d}). \end{array}$$

See Appendix A.

A minor technical detail is our need for an interpolation operator not into ${\bar{V}}_{h}^{f}$ but into ${\bar{V}}_{h}$. It can be obtained by simply interpolating into ${\bar{V}}_{h}^{f}$ and then zeroing out degrees of freedom on $\Gamma_{D}$ via

$$\begin{array}{ll} \pi_{0}:{\bar{V}}_{h}^{f}\to {\bar{V}}_{h}\;\text{defined by}\;\pi_{0}{ū}_{h}(p)=\left\{\begin{array}{cc} 0\; & p\in {\bar{\Gamma }}_{D}\\ {ū}_{h}(p)\; & \text{else} \end{array}\right.\;\text{for}\;p\in 𝒱. & \end{array}$$

Lemma 2 For $ℐ:H^{2}({𝒯}_{h})\to {\bar{V}}_{h}:u\to \pi_{0}{ℐ}_{f}u$ there holds

(17) $$\begin{array}{ll} \underset{T\in {𝒯}_{h}}{\sum }h^{-2}\|u & -ℐu{\|}_{T}^{2}+\|\nabla (u-ℐu){\|}_{T}^{2}≲\underset{T\in {𝒯}_{h}}{\sum }\|\varepsilon (u){\|}_{T}^{2}+\underset{F\in {ℱ}_{h}^{0}}{\sum }h^{-1}\|\Pi_{F}^{R}⟦u⟧{\|}_{F}^{2}\;\forall u\in H^{2}({𝒯}_{h},{\mathbb{R}}^{d}). \end{array}$$

See Appendix A.

#### 3.1.2 Trace norms

For $F\in {ℱ}_{h}$ and an arbitrary element $T\in {𝒯}_{h}$ with $F\in {ℱ}_{T}$ we define for all $û\in {\mathbb{P}}^{k}(F,\mathbb{R})$ discrete versions of the $H^{1/2}(F,\mathbb{R})$ and the $H_{00}^{1/2}(F,\mathbb{R})$‐norm for (scalar) HDG spaces as

‖û‖1,F2:=infw∈ℙk(T){‖∇w‖T2+‖w−û‖j,F2},and‖û‖1,F,02:=infw∈ℙk(T)‖∇w‖T2+‖w−û‖j,F2+∑F˜∈ℱT∖{F}‖w‖j,F2.

In Reference 20 the authors proved the inverse estimate

(18) $$\begin{array}{ll} \|û{\|}_{1,F,0}^{2}≲{\left(\log k\right)}^{3}\|û{\|}_{1,F}^{2}\;\forall û\in {\mathbb{P}}^{k}(F,\mathbb{R})\;\text{such that}\;\Pi_{F}^{0}\hat{v}=0. & \end{array}$$

A similar estimate can be derived for the hybrid, vector‐valued velocity space ${𝒱}_{h}$ and norms involving the symmetric gradient,

(19) ‖(u,û)‖ε,F2:=infw∈ℙk(T)wn=unonF{‖ε(w)‖T2+‖Πk−1(w−û)t‖j,F2}

(20) ‖(u,û)‖ε,F,02:=infw∈ℙk(T)wn=unonF,wn=0on∂T∖F‖ε(w)‖T2+‖Πk−1(w−û)t‖j,F2+∑F˜∈ℱT∖{F}‖Πk−1wt‖j,F˜2.

The difference lies not only in the appearance of ε instead of ∇ but also, and more importantly, in the fact that, as $V_{h}\subseteq H(\text{div})$, the normal trace is enforced strongly, and one has to slightly modify the strategy from Reference 20.

Corollary 1 For $(u,û)\in {𝒱}_{h}$ with $\Pi_{F}^{R}(u_{n}n+{û}_{t})=0$ there holds

(21) $$\begin{array}{ll} \|(u,û){\|}_{\varepsilon ,F,0}^{2}≲{\left(\log k\right)}^{3}\|(u,û){\|}_{\varepsilon ,F}^{2}. & \end{array}$$

See Appendix B.

## 4 THE MCS METHOD

The method considered in this work is based on formulation (2), where ω is used as a Lagrange multiplier to weakly enforce the symmetry constraint (2c), see also References 1, 2, 3. In Reference 7, a novel variational formulation of (2) without the symmetry constraint was presented where the velocity and pressure spaces were H(div,Ω) and $L^{2}(\Omega )$ and the stress space for the variable σ was defined as $H(\text{curl}\;\text{div}):=\{\sigma \in L^{2}(\Omega ,𝔻):\text{div}(\sigma )\in H{\left(\text{div},\Omega \right)}^{*}\}$, where the superscript ∗ denotes the classical dual space. The variational version of (2b) then became

(22) $$\begin{array}{ll} {\left\langle \text{div}(\sigma ),v\right\rangle }_{\text{div}}+(\text{div}(v),p)=(f,v)\;\forall v\in H(\text{div},\Omega ), & \end{array}$$

where ${\left\langle \cdot ,\cdot \right\rangle }_{\text{div}}$ denotes the duality pairing on H(div,Ω). The authors showed that Finite Element approximation of σ in H(curldiv) demands **normal‐tangential** continuity. The method described in the following is based on this variational formulation and in many ways is a variation of previous MCS methods from Reference 7, 8, 9, 22. Like in the method from Reference 9, we incorporate the normal‐tangential continuity of $\sigma_{h}$ via a Lagrange multiplier in ${\hat{V}}_{h}$, similar to approaches taken in hybridized mixed methods for the Poisson problem, see Reference 23, 24, 25. For a detailed discussion on this hybridization technique see also section 7.2.2 in Reference 19. The main motivation for breaking the normal‐tangential continuity by hybridization is that it enables local, element‐wise elimination, or static condensation, of all $\sum_{h}$ and $W_{h}$ dofs. The resulting, condensed, system is the one we actually have to solve and are interested in preconditioning. It will therefore be discussed in great detail in Section 4.2.

The *hybridized mass conserving stress‐yielding method with weakly imposed symmetry* finds $(\sigma_{h},(u_{h},{û}_{h},\omega_{h}),p_{h})\in \sum_{h}\times U_{h}\times Q_{h}$ such that

(23a) $$\begin{array}{llll} \;-\;\nu^{-1}(\sigma_{h},\tau_{h})+b(\tau_{h},(u_{h},{û}_{h},\omega_{h})) & =0 & & \;\forall \tau_{h}\in \sum_{h}, \end{array}$$

(23b) $$\begin{array}{llll} b(\sigma_{h},(v_{h},{\hat{v}}_{h},\eta_{h}))+\nu d^{-1}(\text{div}(u_{h}),\text{div}(v_{h}))-(\text{div}(v_{h}),p_{h}) & =(f,v_{h})\; & & \;\forall (v_{h},{\hat{v}}_{h},\eta_{h})\in U_{h}, \end{array}$$

(23c) $$\begin{array}{llll} \;-\;(\text{div}(u_{h}),q_{h}) & =0\; & & \;\forall q_{h}\in Q_{h}, \end{array}$$

with the bilinear form

$$\begin{array}{llll} b(\tau_{h},(u_{h},{û}_{h},\omega_{h})) & :=\underset{T\in {𝒯}_{h}}{\sum }\int_{T}\text{div}(\tau_{h})\cdot u_{h}-\int_{\partial T}{\left(\tau_{h}\right)}_{nn}{\left(u_{h}\right)}_{n}+\int_{T}\tau_{h}:\omega_{h}-\int_{\partial T}{\left(\tau_{h}\right)}_{nt}{û}_{h}.\; & & \end{array}$$

The first two integrals in b can be interpreted as a discrete version of the duality pair given in (22) and the third weakly enforces the symmetry constraint. The last terms incorporate the normal‐tangential continuity of $\sigma_{h}$ and the tangential part of (2f). Since

$$\begin{array}{ll} \underset{T\in {𝒯}_{h}}{\sum }-\int_{\partial T}{\left(\tau_{h}\right)}_{nt}{û}_{h}=\underset{F\in {ℱ}_{h}}{\sum }\int_{F}⟦{\left(\tau_{h}\right)}_{nt}⟧{û}_{h}, & \end{array}$$

and $⟦{\left(\tau_{h}\right)}_{nt}⟧\in {\hat{V}}_{h}$, testing (23b) with $\left(0,{\hat{v}}_{h},0\right)$, and all ${\hat{v}}_{h}\in {\hat{V}}_{h}$ results in $⟦{\left(\sigma_{h}\right)}_{nt}⟧=0$ on all $F\in {ℱ}_{h}^{0}$. On $\Gamma_{D}$ and $\Gamma_{Ñ}$ the integrals vanish together with ${\left({\hat{v}}_{h}\right)}_{t}=0$ and on $\Gamma_{N}$ the remaining integrals weakly incorporate the tangential part of (2f), ${\left((-\nu \sigma_{h}+p_{h}I)n\right)}_{t}={\left(-\nu \sigma_{h}\right)}_{nt}=0$. For more details on boundary conditions see Remark 2.

We also define the sub problem where we leave out the divergence constraint and solve only for the velocity and stress: Find $(\sigma_{h},(u_{h},{û}_{h},\omega_{h}))\in \sum_{h}\times U_{h}$ such that

(24) $$\begin{array}{ll} 𝒦((\sigma_{h},(u_{h},{û}_{h},\omega_{h})), & (\tau_{h},(v_{h},{\hat{v}}_{h},\eta_{h})))=(f,v_{h})\;\forall (\tau_{h},(v_{h},{\hat{v}}_{h},\eta_{h}))\in \sum_{h}\times U_{h}, \end{array}$$

with

$$\begin{array}{llll} 𝒦((\sigma_{h}, & (u_{h},{û}_{h},\omega_{h})),(\tau_{h},(v_{h},{\hat{v}}_{h},\eta_{h})))=-\nu^{-1}(\sigma_{h},\tau_{h})+b(\tau_{h},(u_{h},{û}_{h},\omega_{h}))+b(\sigma_{h},(v_{h},{\hat{v}}_{h},\eta_{h}))+\nu d^{-1}(\text{div}(u_{h}),\text{div}(v_{h})).\; & & \end{array}$$

Note the addition of the term $\nu d^{-1}(\text{div}(u_{h}),\text{div}(v_{h}))$ in (23b) which is not present in any of the previous MCS methods. It is consistent since the solution is exactly divergence‐free (by (23c) and $\text{div}(V_{h})=Q_{h}$) and guarantees the inf‐sup stability of 𝒦 by compensating for the missing trace of $\sigma_{h}$ which can only approximate the deviatoric part of ε(u). The choice of the constant $\nu d^{-1}$ is motivated by the identity $\varepsilon (u)=\text{dev}(\varepsilon (u))+d^{-1}\text{div}(u)I$. It makes (24) a discretization of −div(νε(u))=f and the velocity Schur complement introduced in Section 4.2 equivalent to a linear elasticity problem, see (31) in the proof of Lemma 5. We want to stress again that preconditioners for such problems (although only for conforming methods) are standard in the literature and widely available. A different choice of constant would be possible, but changes the norm represented by the velocity Schur complement. For example, a large (penalty) parameter $\varepsilon^{-1}\nu$ (i.e., with a small ε>0) instead of $\nu d^{-1}$ would be a valid choice but would make the resulting velocity Schur complement extremely difficult to precondition as it then has the divergence constraint essentially built in.

Remark 1 The MCS method here, like the one from Reference 8 to which it is most closely related to, features insufficient inter‐element coupling for the lowest order case k=1 and is only stable for k≥2. This is discussed in detail in Reference 9, where a stable minimal order MCS method for k=1 was introduced. We treat the lowest order case separately in Section 7.

Remark 2 Reference 7 contains an exhaustive discussion of possible boundary conditions for the standard MCS method. One type of boundary condition for which it is particularly suitable are non‐homogenous viscous stress ones, where ${\left(\sigma_{h}\right)}_{nt}=g_{t}$ can be demanded strongly for a given tangential traction $g_{t}$, and is incorporated as an essential boundary condition in $\sum_{h}$. In the hybridized MCS method, where the normal‐tangential continuity in $\sum_{h}$ is no longer given, this boundary condition is instead realized by an additional right‐hand side term $\int_{\Gamma_{N}}-g_{t}{\hat{v}}_{h}$ in (23). Since ${\left(\sigma_{h}\right)}_{nt}\in {\hat{V}}_{h}$, this still strongly yields ${\left(\sigma_{h}\right)}_{nt}=g_{t}$, as testing (23b) with $\left(0,{\hat{v}}_{h},0\right)$ shows.

### 4.1 Stability analysis

In the following we summarize the stability results for the discrete method defined above. We only prove solvability of (24), all other results follow with the same techniques and steps as in References 7, 8, 9, 22. Lemma 4, which can, just as Lemma 3, be found in the stated literature, is an inf‐sup stability result for the constraint given by the bilinear form b. It is posed in the semi‐norm $|\cdot {|}_{U_{h},*}$ as, since all elements in $\sum_{h}$ are trace‐free, the divergence of functions in $U_{h}$ can not be controlled. Theorem 1 states that with the addition of the term $\nu d^{-1}(\text{div}(u_{h}),\text{div}(v_{h}))$ in (23b) we can switch to the proper norm $\|\cdot {\|}_{U_{h}}$ and (24) is solvable independently of the divergence constraint. Finally, Corollary 2, which is again already proven in the literature, provides solvability of (23) including the divergence constraint. Note that the stability analysis in this section is only robust in terms of the mesh size h. Particularly the inf‐sup constant in Corollary 2 and Theorem 1 is not known to be independent of the polynomial order k.

Lemma 3 There hold the continuity estimates

$$\begin{array}{llllllll} \nu^{-1}(\sigma_{h},\tau_{h}) & \leq \nu^{-1}\|\sigma_{h}{\|}_{0}\|\tau_{h}{\|}_{0}\; & & \forall \tau_{h},\sigma_{h}\in \sum_{h},\; & & \; & & \\ b(\tau_{h},(u_{h},{û}_{h},\omega_{h})) & ≲\|\tau_{h}{\|}_{0}\|(u_{h},{û}_{h},\omega_{h}){\|}_{U_{h}}\; & & \forall (\tau_{h},(u_{h},{û}_{h},\omega_{h}))\in \sum_{h}\times U_{h},\; & & \; & & \\ \left(\text{div}(u_{h}),q_{h}\right) & ≲\|(u_{h},{û}_{h},\omega_{h}){\|}_{U_{h}}\|q_{h}{\|}_{0}\; & & \forall (q_{h},(u_{h},{û}_{h},\omega_{h}))\in Q_{h}\times U_{h},\; & & \; & & \\ \nu d^{-1}(\text{div}(u_{h}),\text{div}(v_{h})) & \leq \nu d^{-1}\|\text{div}(u_{h}){\|}_{0}\|\text{div}(v_{h}){\|}_{0}\; & & \forall u_{h},v_{h}\in V_{h}.\; & & \; & & \end{array}$$

Lemma 4 Let $(v_{h},{\hat{v}}_{h},\eta_{h})\in U_{h}$ be arbitrary. There exists a $\sigma_{h}\in \sum_{h}$ such that

$$\begin{array}{ll} b(\sigma_{h},(v_{h},{\hat{v}}_{h},\eta_{h}))≳|(v_{h},{\hat{v}}_{h},\eta_{h}){|}_{U_{h},*}^{2},\;\text{and}\;\|\sigma_{h}{\|}_{0}≲|(v_{h},{\hat{v}}_{h},\eta_{h}){|}_{U_{h},*}. & \end{array}$$

Theorem 1 Let $(\tau_{h},(v_{h},{\hat{v}}_{h},\eta_{h}))\in \sum_{h}\times U_{h}$ be arbitrary, there holds the inf‐sup stability

supσh∈∑h(uh,ûh,ηh)∈Uh𝒦((σh,(uh,ûh,ωh)),(τh,(vh,v^h,ηh)))ν−1/2‖σh‖0+ν1/2‖(uh,ûh,ωh)‖Uh≳ν−1/2‖τh‖0+ν1/2‖(vh,v^h,ηh)‖Uh.

This follows with standard techniques, that is, using Lemma 4, Young's and Cauchy Schwarz's inequality and the norm equivalence (13).

Corollary 2 Let $(\tau_{h},(v_{h},{\hat{v}}_{h},\eta_{h}),q_{h})\in \sum_{h}\times U_{h}\in Q_{h}$ be arbitrary, there holds the inf‐sup stability

supσh∈∑h(uh,ûh,ηh)∈Uhph∈Qh𝒦((σh,(uh,ûh,ωh)),(τh,(vh,v^h,ηh)))+(div(uh),qh)+(div(vh),ph)ν−1/2(‖σh‖0+‖ph‖0)+ν1/2‖(uh,ûh,ωh)‖Uh≳(ν−1/2‖τh‖0+‖qh‖0)+ν1/2‖(vh,v^h,ηh)‖Uh.

For a smooth exact solution we can further derive the following optimal error estimate.

Corollary 3 Let $u\in H^{m}({𝒯}_{h},{\mathbb{R}}^{d})\cap H^{1}(\Omega ,{\mathbb{R}}^{d})$, $\sigma \in H^{m-1}({𝒯}_{h},𝔻)\cap H^{1}(\Omega ,𝔻)$, $\omega \in H^{m-1}({𝒯}_{h},𝕂)\cap L^{2}(\Omega ,𝕂)$, and $p\in H^{m-1}({𝒯}_{h},\mathbb{R})\cap L^{2}(\Omega ,\mathbb{R})$ be the exact solution of the Stokes problem (2) and set $û=u{|}_{F}$ for all $F\in {ℱ}_{h}$. Let $(\sigma_{h},(u_{h},{û}_{h},\omega_{h}))\in \sum_{h}\times U_{h}$ be the solution of (23) and let s=min(m−1,k), then there holds the error estimate

$$\begin{array}{llll} \nu^{-1}(\|\sigma -\sigma_{h}{\|}_{0}+\|p-p_{h}{\|}_{0}) & +\|(u-u_{h},û-{û}_{h},\omega -\omega_{h}){\|}_{U_{h}}\; & & \\ & ≲h^{s}(\nu^{-1}(\|\sigma {\|}_{H^{s}({𝒯}_{h},𝔻)}+\|p{\|}_{H^{s}({𝒯}_{h},\mathbb{R})})+\|u{\|}_{H^{s+1}({𝒯}_{h},{\mathbb{R}}^{d})}+\|\omega {\|}_{H^{s}({𝒯}_{h},𝕂)}).\; & & \end{array}$$

### 4.2 Static condensation of local variables

We now discuss the structure of the Finite Element matrix directly obtained from the MCS method (23) and that of various Schur complements thereof. Writing $\phi^{\sigma },\phi^{u},\phi^{û},\phi^{\omega }$ and $\phi^{p}$ for the basis functions of $\sum_{h},V_{h},{\hat{V}}_{h},W_{h}$ and $Q_{h}$ respectively and, complying with the notation for the Galerkin isomorphism introduced in Section 2, u for the coefficients of $u_{h}$ with respect to the basis given by $\phi^{u}$, and so forth, (23) in matrix form is

(25) $$\begin{array}{ll} \left(\begin{array}{ccc} \left(\begin{array}{cc} -\;M_{\sigma \sigma } & B_{\omega \sigma }^{T}\\ B_{\omega \sigma } & 0 \end{array}\right) & \left(\begin{array}{cc} B_{u\sigma }^{T} & B_{û\sigma }^{T}\\ 0 & 0 \end{array}\right) & \left(\begin{array}{c} 0\\ 0 \end{array}\right)\\ \left(\begin{array}{cc} B_{u\sigma } & 0\\ B_{û\sigma } & 0 \end{array}\right) & \left(\begin{array}{cc} A_{uu}^{\text{div}} & 0\\ 0 & 0 \end{array}\right) & \left(\begin{array}{c} B_{pu}^{T}\\ 0 \end{array}\right)\\ \left(\begin{array}{cc} 0 & 0 \end{array}\right) & \left(\begin{array}{cc} B_{pu} & 0 \end{array}\right) & 0 \end{array}\right)\left(\begin{array}{c} \left(\begin{array}{c} \sigma \\ \omega \end{array}\right)\\ \left(\begin{array}{c} u\\ û \end{array}\right)\\ p \end{array}\right)=\left(\begin{array}{c} \left(\begin{array}{c} 0\\ 0 \end{array}\right)\\ \left(\begin{array}{c} F\\ 0 \end{array}\right)\\ 0 \end{array}\right). & \end{array}$$

The right hand side vector F is given by $F_{i}=(f,\phi_{i}^{u})$. The system matrix with its entries

$$\begin{array}{llllllll} {\left(M_{\sigma \sigma }\right)}_{ij} & =\nu^{-1}(\phi_{i}^{\sigma },\phi_{j}^{\sigma }),\; & {\left(B_{û\sigma }\right)}_{ij} & =\underset{T}{\sum }-\int_{\partial T}{\left(\phi_{j}^{\sigma }\right)}_{nt}{\left(\phi_{i}^{û}\right)}_{t},\; & & \; & & \\ {\left(B_{\omega \sigma }\right)}_{ij} & =\underset{T}{\sum }\int_{\partial T}\phi_{j}^{\sigma }:\phi_{i}^{\omega },\; & {\left(A_{uu}^{\text{div}}\right)}_{ij} & =\nu d^{-1}(\text{div}(\phi_{i}^{u}),\text{div}(\phi_{j}^{u})),\; & & \; & & \\ {\left(B_{pu}\right)}_{ij} & =(\text{div}(\phi_{j}^{u}),\phi_{i}^{p}),\; & {\left(B_{u\sigma }\right)}_{ij} & =\underset{T}{\sum }\int_{T}\text{div}(\phi_{j}^{\sigma })\phi_{i}^{u}-\int_{\partial T}{\left(\phi_{j}^{\sigma }\right)}_{nn}{\left(\phi_{i}^{u}\right)}_{n}.\; & & \; & & \end{array}$$

is a saddle point matrix with Lagrange multipliers ω,u,û and p enforcing (2c), (2a), the nt‐continuity of σ, and (2d) respectively.

**Static condensation of** σ,ω

The diagonal block for σ,ω does not couple with the pressure via the incompressibility constraint and, thanks to the introduction of ${û}_{h}$ as additional multiplier, is block diagonal. It is also invertible since every block represents the simple projection problem of finding $\left(\sigma_{h}^{T},\omega_{h}^{T})\in \sum_{h}(T)\times W_{h}(T\right)$ for some $T\in {𝒯}_{h}$ such that

(26a) $$\begin{array}{ll} -\;\frac{1}{\nu }{\left(\sigma_{h}^{T},\tau_{h}\right)}_{T}+{\left(\tau_{h},\omega_{h}^{T}\right)}_{T} & =g_{T}(\tau_{h})\;\forall \tau_{h}\in \sum_{h}(T), \end{array}$$

(26b) $$\begin{array}{ll} \;{\left(\sigma_{h}^{T},\eta_{h}\right)}_{T} & =0\;\forall \eta_{h}\in W_{h}(T), \end{array}$$

where $\sum_{h}(T),W_{h}(T)$ are the restrictions of the corresponding (discontinuous) global spaces to T and $g_{T}$ is some right hand side. Standard arguments and the Brezzi theorem prove that (26) is inf‐sup stable, that is, writing

$$\begin{array}{ll} M:=\left(\begin{array}{cc} -\;M_{\sigma \sigma } & B_{\omega \sigma }^{T}\\ B_{\omega \sigma } & 0 \end{array}\right),\;B_{\sigma }:=\left(\begin{array}{cc} B_{u\sigma } & 0\\ B_{û\sigma } & 0 \end{array}\right),\;A^{\text{div}}:=\left(\begin{array}{cc} A_{uu}^{\text{div}} & 0\\ 0 & 0 \end{array}\right), & \end{array}$$

M is invertible and the Schur complement $A:=A^{\text{div}}-B_{\sigma }M^{-1}B_{\sigma }^{T}$ is well defined and, as M is block diagonal, can be computed element‐wise. Eliminating σ,ω from (25) in this way leaves us with the system

(27) $$\begin{array}{ll} K\left(\begin{array}{c} \left(\begin{array}{c} u\\ û \end{array}\right)\\ p \end{array}\right):=\left(\begin{array}{cc} A & B^{T}\\ B & 0 \end{array}\right)\left(\begin{array}{c} \left(\begin{array}{c} u\\ û \end{array}\right)\\ p \end{array}\right)=\left(\begin{array}{c} \left(\begin{array}{c} F\\ 0 \end{array}\right)\\ 0 \end{array}\right). & \end{array}$$

The symmetry of K is obvious and in the next Lemma 5 we show that the upper left block A is also positive definite and we are now in the very standard setting of a saddle point problem with symmetric and positive definite (SPD) “A‐block”. The velocity unknowns $u_{h},{û}_{h}$ move to the position of primal variables, while the pressure $p_{h}$ remains the Lagrange parameter for the divergence constraint. After solving (27) to get $u_{h},{û}_{h},p_{h}$, we can recover $\sigma_{h}$ and $\omega_{h}$ by solving the local problems (26).

Lemma 5 The Schur complement A is symmetric positive definite and with

$$\begin{array}{ll} {𝒱}_{h}^{c}:=\{(v_{h},{\hat{v}}_{h})\in {𝒱}_{h}:v_{h}\in H^{1}(\Omega ,{\mathbb{R}}^{d}),\Pi_{F}^{k-1}{\left(u_{h}-{û}_{h}\right)}_{t}=0\;\forall F\in {ℱ}_{h}\} & \end{array}$$

there holds

(28) $$\begin{array}{ll} \nu \|(u_{h},{û}_{h}){\|}_{\varepsilon ,h}^{2}≲\|(u_{h} & ,{û}_{h}){\|}_{A}^{2}\leq \nu \|(u_{h},{û}_{h}){\|}_{\varepsilon ,h}^{2}\;\forall (u_{h},{û}_{h})\in {𝒱}_{h}, \end{array}$$

(29) $$\begin{array}{ll} \|(u_{h},{û}_{h}){\|}_{A}^{2} & =\nu \|\varepsilon (u_{h}){\|}_{0}^{2}\;\forall (u_{h},{û}_{h})\in {𝒱}_{h}^{c}. \end{array}$$

Let $(u_{h},{û}_{h})\in {𝒱}_{h}$ be arbitrary and set $\left(\sigma ,\omega ):=-M^{-1}B_{\sigma }^{T}(u,û\right)$, that is, the local functions $\left(\sigma_{h|T},\omega_{h|T}):=(\sigma_{h}^{T},\omega_{h}^{T}\right)$ are the solution of (26) with right hand side

(30) $$\begin{array}{ll} g_{T}(\tau_{h}):=-b(\tau_{h},(u_{h},{û}_{h},0))=\int_{T}\tau_{h}:\nabla u_{h}-\int_{\partial T}{\left(\tau_{h}\right)}_{nt}{\left(u_{h}-{û}_{h}\right)}_{t}, & \end{array}$$

where we used an element‐wise integration by parts for b. From (26b) we see $(B_{\omega \sigma }\sigma ,\omega )=\int_{\Omega }\sigma_{h}:\omega_{h}=0$ and there holds

$$\begin{array}{llll} \|(u_{h},{û}_{h}){\|}_{A}^{2} & =(\sigma ,\omega ,u,û)\left(\begin{array}{cc} M & B_{\sigma }^{T}\\ B_{\sigma } & A^{\text{div}} \end{array}\right){\left(\sigma ,\omega ,u,û\right)}^{T}\; & & \\ & =(M_{\sigma \sigma }\sigma ,\sigma )+(A^{\text{div}}(u,û),(u,û)).\; & & \end{array}$$

With $(A^{\text{div}}(u,û),(u,û))=\nu d^{-1}\|\text{div}(u_{h}){\|}_{0}^{2}$ this gives

(31) $$\begin{array}{ll} \|(u_{h},{û}_{h}){\|}_{A}^{2}=\nu^{-1}\|\sigma_{h}{\|}_{0}^{2}+\nu d^{-1}\|\text{div}(u_{h}){\|}_{0}^{2}. & \end{array}$$

We now insert (30) into (26a) and test with $\tau_{h}=\sigma_{h}^{T}$. The term ${\left(\tau_{h},\omega_{h}^{T}\right)}_{T}=0$ drops out due to (26b) and we see that $\forall T\in {𝒯}_{h}$

$$\begin{array}{ll} \nu^{-1}\|\sigma_{h}{\|}_{T}^{2}=g_{T}(\sigma_{h}). & \end{array}$$

We can use (26b) again to see ${\left(\sigma_{h},\nabla u_{h}\right)}_{T}={\left(\sigma_{h},\text{dev}(\varepsilon (u_{h}))\right)}_{T}$, as $\text{tr}(\sigma_{h})=0$ is built into $\sum_{h}(T)$ and get

$$\begin{array}{llll} \nu^{-1}\|\sigma_{h}{\|}_{T} & \leq \frac{\left|{\left(\nabla u_{h},\sigma_{h}\right)}_{T}|+|{\left({\left(\sigma_{h}\right)}_{nt},{\left(u_{h}-{û}_{h}\right)}_{t}\right)}_{\partial T}\right|}{\|\sigma_{h}{\|}_{T}}\; & & \\ & =\frac{\left|{\left(\text{dev}(\varepsilon (u_{h})),\sigma_{h}\right)}_{T}|+\sum_{F\in {ℱ}_{T}}|{\left({\left(\sigma_{h}\right)}_{nt},{\left(u_{h}-{û}_{h}\right)}_{t}\right)}_{F}\right|}{\|\sigma_{h}{\|}_{T}}\; & & \\ & \leq \|\text{dev}(\varepsilon (u_{h})){\|}_{T}+\underset{F\in {ℱ}_{T}}{\sum }\underset{\tau_{h}\in \sum_{h}(T)}{\sup}\frac{{\left({\left(\tau_{h}\right)}_{nt},{\left(u_{h}-{û}_{h}\right)}_{t}\right)}_{F}}{\|\tau_{h}{\|}_{T}}\; & & \\ & \leq \|\text{dev}(\varepsilon (u_{h})){\|}_{T}+\underset{F\in {ℱ}_{T}}{\sum }\|\Pi^{k-1}{\left(u_{h}-{û}_{h}\right)}_{t}{\|}_{j,F}.\; & & \end{array}$$

Thus, with (31)

$$\begin{array}{llll} \|(u_{h},{û}_{h}){\|}_{A}^{2} & \leq \nu \underset{T\in {𝒯}_{h}}{\sum }(\|\text{dev}(\varepsilon (u_{h})){\|}_{T}^{2}+d^{-1}\|\text{div}(u_{h}){\|}_{T}^{2}+\underset{F\in {ℱ}_{T}}{\sum }\|\Pi^{k-1}{\left(u_{h}-{û}_{h}\right)}_{t}{\|}_{j,F}^{2})\; & & \\ & =\nu \underset{T\in {𝒯}_{h}}{\sum }(\|\varepsilon (u_{h}){\|}_{T}^{2}+\underset{F\in {ℱ}_{T}}{\sum }\|\Pi^{k-1}{\left(u_{h}-{û}_{h}\right)}_{t}{\|}_{j,F}^{2})=\nu \|(u_{h},{û}_{h}){\|}_{\varepsilon ,h}^{2}.\; & & \end{array}$$

It remains to prove the other direction. By Lemma 4 there exists a $\tau_{h}\in \sum_{h}$ with $\|\tau {\|}_{0}≲|u_{h},{û}_{h},\omega_{h}{|}_{U_{h},*}$ such that, once again inserting (30) into (26a), we see that

$$\begin{array}{ll} |(u_{h},{û}_{h},\omega_{h}){|}_{U_{h},*}≲\frac{b(\tau_{h},(u_{h},{û}_{h},\omega_{h}))}{\|\tau_{h}{\|}_{0}}=\underset{T\in {𝒯}_{h}}{\sum }\frac{\nu^{-1}{\left(\sigma_{h},\tau_{h}\right)}_{T}}{\|\tau_{h}{\|}_{0}}\leq \nu^{-1}\|\sigma_{h}{\|}_{0} & \end{array}$$

and therefore there holds

$$\begin{array}{llll} \nu \|(u_{h},{û}_{h}){\|}_{\varepsilon ,h}^{2} & ≲\nu |(u_{h},{û}_{h},\omega_{h}){|}_{U_{h},*}^{2}+\nu d^{-1}\|\text{div}(u_{h}){\|}_{0}^{2}≲\nu^{-1}\|\sigma_{h}{\|}_{0}^{2}+\nu d^{-1}\|\text{div}(u_{h}){\|}_{0}^{2}=\|(u_{h},{û}_{h}){\|}_{A}^{2}.\; & & \end{array}$$

Finally, for $(u_{h},{û}_{h})\in {𝒱}_{h}^{c}$ we have $\Pi_{F}^{k-1}{\left(u_{h}-{û}_{h}\right)}_{t}=0\;\forall F\in {ℱ}_{h}$ and the distributional terms in (30) vanish. The solutions of (26) are then simply given by $\sigma_{h}=-\nu \text{dev}(\varepsilon (u_{h}))$ and $\omega_{h}=\kappa (\text{curl}(u_{h}))$ and (31) states

$$\begin{array}{llll} \|(u_{h},{û}_{h}){\|}_{A}^{2} & =\nu \|\text{dev}(\varepsilon (u_{h})){\|}_{0}^{2}+\nu d^{-1}\|\text{div}(u_{h}){\|}_{0}^{2}=\nu \|\varepsilon (u_{h}){\|}_{0}^{2}.\; & & \end{array}$$

Remark 3 In A, we have a discretization of div(νε(u)) with degrees of freedom $u_{h}$ and ${û}_{h}$ only. This is less reminiscent of a mixed method like MCS than of a HDG method and it is interesting to further elaborate on the relationship between the MCS method and DG and HDG methods. In general, DG and HDG methods require a stabilizing term to assure solvability. An example is the well known interior penalty method where the $L^{2}$‐norm of jumps, $\alpha \frac{k^{2}}{h}\|u_{h}-{û}_{h}{\|}_{F}^{2}$ for $F\in {ℱ}_{h}$ with some sufficiently large α is used. Any dependence on such a parameter is avoided here, however this is not an unique feature of the MCS method. Other DG and HDG methods that also avoid this parameter feature a lifting $\sigma_{h}$ of the jump similar to (9) instead of its $L^{2}$ norm, see Reference 5. That lifting has to be explicitly computed and is then condense out. A final class of DG methods, for example the one in Reference 26, see also References 5, 27, features a simultaneous lifting of the jump and the fluxes. This is similar to what happens here, where $\sigma_{h}$ both approximates the flux −νε(u) and *automatically and canonically* stabilizes the condensed system through its interaction with the tangential jumps.

**Static condensation of high order velocity functions**

The basis functions of $V_{h}$ can be split into two different types, see Reference 28. We write $\phi^{u,\circ }$ for the high order “element bubble” basis functions whose support is entirely within some element $T\in {𝒯}_{h}$ and whose normal trace on ∂T vanishes. The span of these basis functions is denoted by $V_{h}^{\circ }$ and we write ${𝒱}_{h}^{\circ }:=V_{h}^{\circ }\times \{0\}\subseteq {𝒱}_{h}$. The remaining basis functions $\phi^{u,\partial }$ of $V_{h}$ have support entirely within the patch of some facet F and their normal trace on all other facets in the patch vanishes. As the supports of $\phi^{u,\circ }$ belonging to different elements do not overlap, in

$$\begin{array}{ll} A=\left(\begin{array}{ccc} A_{\circ \circ } & A_{\circ \partial } & A_{\circ û}\\ A_{\partial \circ } & A_{\partial \partial } & A_{\partial û}\\ A_{û\circ } & A_{û\partial } & A_{ûû} \end{array}\right), & \end{array}$$

the upper left block $A_{\circ \circ }$ is block diagonal and invertible. This lets us form a second, “double” Schur complement

$$\begin{array}{ll} A^{\partial }:=\left(\begin{array}{cc} A_{\partial \partial } & A_{\partial û}\\ A_{û\partial } & A_{ûû} \end{array}\right)-\left(\begin{array}{c} A_{\partial \circ }\\ A_{û\circ } \end{array}\right)A_{\circ \circ }^{-1}\left(\begin{array}{cc} A_{\circ \partial } & A_{\circ û} \end{array}\right). & \end{array}$$

In the bigger system (27), all $V_{h}$ degrees of freedom couple with the divergence constraint and we cannot perform this static condensation independently of the pressure variables. However, for higher order problems, implementing multiplication with A via the exact factorization

(32) $$\begin{array}{ll} A & =\left(\begin{array}{cc} I & A_{\partial \circ }A_{\circ \circ }^{-1}\\ 0 & I \end{array}\right)\left(\begin{array}{cc} A^{\partial } & 0\\ 0 & A_{\circ \circ } \end{array}\right)\left(\begin{array}{cc} I & 0\\ A_{\circ \circ }^{-1}A_{\circ \partial } & I \end{array}\right), \end{array}$$

is still advantageous. Both the left and right factors as well as $A_{\circ \circ }$ are block diagonal and only $A^{\partial }$ instead of the larger A needs to be assembled as a proper sparse matrix. We will revisit the idea of also preconditioning A via this factorization in Section 6.1.

Splitting the coordinate vector u of the $V_{h}$ component of $(u_{h},{û}_{h})\in {𝒱}_{h}$ into $u_{\circ }$ and $u_{\partial }$, the norm induced by $A^{\partial }$ on $\left(u_{\partial },û\right)$ is

(33) $$\begin{array}{ll} \|(u_{\partial },û){\|}_{A^{\partial }}=\underset{v_{\circ }}{\inf}\|(u_{\circ }+v_{\circ },u_{\partial },û){\|}_{A}=\underset{(v_{h},{\hat{v}}_{h})\in {𝒱}_{h}^{\circ }}{\inf}\|(u_{h}+v_{h},{û}_{h}+{\hat{v}}_{h}){\|}_{A}. & \end{array}$$

That is, the norm induced by $A^{\partial }$ is just the one induced by A on the energy minimal extension to ${𝒱}_{h}^{\circ }$ dofs. The lifting operator, or (discrete) harmonic extension, $ℋ:{𝒱}_{h}\to {𝒱}_{h}$ maps $\left(u_{h},{û}_{h}\right)$, to the minimizer in (33):

$$\begin{array}{ll} ℋ(u_{h},{û}_{h})=\underset{(v_{h},{\hat{v}}_{h})\in {𝒱}_{h}^{\circ }}{\text{arg min}}\|(u_{h}+v_{h},{û}_{h}+{\hat{v}}_{h}){\|}_{A}. & \end{array}$$

Equivalently, writing $\left(w,ŵ){∼}_{G}(w_{h},{ŵ}_{h}):=ℋ(u_{h},{û}_{h}\right)$, the operator ℋ is defined by

(34) $$\begin{array}{ll} w_{\partial }=u_{\partial },\;\;ŵ=û\;\text{and}\;w_{\circ }=-A_{\circ }^{-1}(A_{\circ \partial }u_{\partial }+A_{\circ û}û). & \end{array}$$

The range of ℋ is

(35) $$\begin{array}{ll} {𝒱}_{h}^{\text{harm}}:=ℋ({𝒱}_{h})=\{(u_{h},{û}_{h})\in {𝒱}_{h}:A_{\circ \circ }u_{\circ }+A_{\circ \partial }u_{\partial }+A_{\circ û}û=0\}, & \end{array}$$

and for such “discrete harmonic” or “lifted” functions $(u_{h},{û}_{h})\in {𝒱}_{h}^{\text{harm}}$ there holds

(36) $$\begin{array}{ll} \|(u_{h},{û}_{h}){\|}_{A}=\|(u_{\circ },u_{\partial },û){\|}_{A}=\|(u_{\partial },û){\|}_{A^{\partial }}. & \end{array}$$

Here we encounter a slight complication of notation: Per default, $(u_{h},{û}_{h})\in {𝒱}_{h}^{\text{harm}}$ is associated with its coordinate vector $\left(u^{\circ },u^{\partial },û\right)$, but $A^{\partial }$ only takes the $\left(u^{\partial },û\right)$ coordinates (which determine $u^{\circ }$ according to (35)). The space ${𝒱}_{h}^{\text{harm}}$ is spanned by lifted, discrete harmonic, basis functions,

$$\begin{array}{ll} {𝒱}_{h}^{\text{harm}}=\text{span}\{ℋ(\phi^{u,\partial },0):\phi^{u,\partial }\in \Phi^{u,\partial }\}+\text{span}\{ℋ(0,\phi^{û}):\phi^{û}\in \Phi^{û}\}, & \end{array}$$

where $\Phi^{u,\partial }$ is the set of all $\phi^{u,\partial }$ and $\Phi^{û}$ the one of all $\phi^{û}$ basis functions. The induced Galerkin Isomorphism ${∼}_{G}^{\partial }$ defines the natural operator $A^{\partial }$ associated with $A^{\partial }$ and identifies $(u_{h},{û}_{h})\in {𝒱}_{h}^{\text{harm}}$ with $\left(u^{\partial },û\right)$. Where there is potential for confusion we explicitly write $\left(u_{h},{û}_{h}){∼}_{G}^{\partial }(u^{\partial },û\right)$ in contrast to $\left(u_{h},{û}_{h}){∼}_{G}(u^{\circ },u^{\partial },û\right)$.

Analogously, we define the Schur complement like norm

(37) $$\begin{array}{ll} \|(u_{h},{û}_{h}){\|}_{\varepsilon ,h,\partial }:=\underset{(v_{h},{\hat{v}}_{h})\in {𝒱}_{h}^{\circ }}{\inf}\|(u_{h}+v_{h},{û}_{h}+{\hat{v}}_{h}){\|}_{\varepsilon ,h} & \end{array}$$

and the associated lifting operator ${ℋ}_{\varepsilon }:{𝒱}_{h}\to {𝒱}_{h}$ such that

$$\begin{array}{ll} \|(u_{h},{û}_{h}){\|}_{\varepsilon ,h,\partial }=\|{ℋ}_{\varepsilon }(u_{h},{û}_{h}){\|}_{\varepsilon ,h,}\;\forall (u_{h},{û}_{h})\in {𝒱}_{h}. & \end{array}$$

Note that both $\|\cdot {\|}_{A^{\partial }}$ and $\|\cdot {\|}_{\varepsilon ,h,\partial }$ can be defined on the entirety of ${𝒱}_{h}$ but are only semi‐norms as they vanish on $\ker ℋ=\ker{ℋ}_{\varepsilon }={𝒱}_{h}^{\circ }$. Restricted to ${𝒱}_{h}^{\text{harm}}$ they are proper norms and equivalent.

Corollary 4 There holds

(38) $$\begin{array}{ll} \gamma^{-1}\nu \|(u_{h},{û}_{h}){\|}_{\varepsilon ,h,\partial }^{2}\leq \|(u_{h},{û}_{h}){\|}_{A^{\partial }}^{2}\leq \nu \|(u_{h},{û}_{h}){\|}_{\varepsilon ,h,\partial }^{2}\;\forall (u_{h},{û}_{h})\in {𝒱}_{h}^{\text{harm}}, & \end{array}$$

for some γ(k)>0 discussed in more detail shortly in Remark 4.

Follows immediately from Lemma 5.

Remark 4 An interesting question is whether, and if so, how strongly, both the constant in the lower bound in (28) and γ depend on k. Note that only the latter constant enters into condition number estimates in Section 6. Numerical experiments on the unit tetrahedron suggest that the former depends linearly on k while there holds γ=𝒪(1) or possibly $\gamma =𝒪(\log{\left(k\right)}^{l})$ with some moderate l>0. We have not further pursued a rigorous proof for this latter, admittedly crucial, fact. Such a proof would essentially require a k‐explicit version of Lemma 4 for functions in ${𝒱}_{h}^{\text{harm}}$.

## 5 PRECONDITIONING FRAMEWORK

A final Schur complement can be formed with respect to the pressure unknowns, however this involves the inverse $A^{-1}$. With the resulting (negative) pressure Schur complement $S_{p}:=BA^{-1}B^{T}$, we have the exact factorization

(39) $$\begin{array}{ll} K=\left(\begin{array}{cc} I & 0\\ BA^{-1} & I \end{array}\right)\left(\begin{array}{cc} A & 0\\ 0 & -S_{p} \end{array}\right)\left(\begin{array}{cc} I & A^{-1}B^{T}\\ 0 & I \end{array}\right) & \end{array}$$

for the saddle point matrix K. Solving (27) could in principle be reduced to solving separate problems for the pressure and velocity. While this is not feasible due to the appearance of $A^{-1}$ in the pressure Schur complement, this line of thought still takes a prominent role in common preconditioning techniques for K based on separate preconditioners $\hat{A}$ for A and ${\hat{S}}_{p}$ for $S_{p}$. See Reference 10 and the references therein for an overview of such methods. Motivated by (39), here we use

(40) $$\begin{array}{ll} {\hat{K}}^{-1}:=\left(\begin{array}{cc} I & -{\hat{A}}^{-1}B^{T}\\ 0 & I \end{array}\right)\left(\begin{array}{cc} {\hat{A}}^{-1} & 0\\ 0 & {\hat{S}}_{p}^{-1} \end{array}\right)\left(\begin{array}{cc} I & 0\\ -\;B{\hat{A}}^{-1} & I \end{array}\right). & \end{array}$$

Note that unlike suggested by (40), the operation $x\mapsto {\hat{K}}^{-1}x$ can be implemented such that it requires only two applications of ${\hat{A}}^{-1}$ instead of three. A rigorous analysis of $\hat{K}$ for the generic saddle point case as well as a number of other, similar, preconditioners built from $\hat{A}$ and ${\hat{S}}_{p}$ can be found in Reference 29.

### 5.1 Pressure Schur preconditioner

From the standard Stokes‐LBB condition on ${𝒱}_{h}$ using the norm $\|\cdot {\|}_{\varepsilon ,h}$, see for example in Reference 9, and the equivalence result Lemma 5, we can conclude the MCS Stokes‐LBB condition

$$\begin{array}{ll} \underset{(v_{h},{\hat{v}}_{h})\in {𝒱}_{h}}{\sup}\frac{\left(\text{div}(v_{h}),q_{h}\right)}{\|(v_{h},{\hat{v}}_{h}){\|}_{A}}\geq \gamma_{L}\|q_{h}{\|}_{0}\;\forall q_{h}\in Q_{h}. & \end{array}$$

with $\gamma_{L}>0$ independent of h. It is generally well known that given this LBB condition, $S_{p}$ is equivalent to the scaled mass matrix $(M_{p}p,q):=\nu^{-1}{\left(p_{h},q_{h}\right)}_{0}$ for $p_{h},q_{h}\in Q_{h}$. The bounds in that equivalence depend on a continuity constant (Lemma 3) and, more importantly, $\gamma_{L}$, see References 11, 30. As $Q_{h}$ is completely discontinuous across elements in the MCS discretization, inverting the block‐diagonal matrix $M_{p}$ is feasible and we use ${\hat{S}}_{p}:=M_{p}$. Since therefore $\gamma_{L}$ is the primary constant determining the quality of the pressure preconditioner, it is very relevant what else it might depend on. For norms similar to the ones used here, $\gamma_{L}$ was proven to be independent of k in two dimensions in Reference 31 and numerical experiments carried out in the same work strongly suggest that the independence also holds in three dimensions. The k‐robustness is further supported by numerical experiments carried out in this work, see Section 8. There remains, however, a dependence of the domain Ω itself, and in fact $\gamma_{L}$ even tends to zero for certain domain shapes, for example, channels with increasingly higher aspect ratios, see Reference 32. This imposes a fundamental limitation on all methods that, like the one here, precondition the velocity problem independently of the divergence constraint.

### 5.2 Auxiliary space preconditioning

We give the fictitious space Lemma 6 below in the compact form it takes, for example, in Reference 14 [Theorem 6.3].

Lemma 6 Let $H,\tilde{H}$ be two real Hilbert spaces equipped with norms induced by $A:H\to H^{*}$ and $\tilde{A}:\tilde{H}\to {\tilde{H}}^{*}$ and let there exist a linear operator $\Pi :\tilde{H}\to H$ such that the continuity condition

(41) $$\begin{array}{ll} {\left\|\Pi \tilde{v}\right\|}_{A}^{2}\leq c_{0}{\left\|\tilde{v}\right\|}_{\tilde{A}}^{2}\;\;\forall \tilde{v}\in \tilde{H}, & \end{array}$$

and the stability condition,

(42) $$\begin{array}{ll} \forall v\in H\;\exists \tilde{v}\in \tilde{H}\;\text{such that}\;v=\Pi \tilde{v}\;\text{and}\;{\left\|\tilde{v}\right\|}_{\tilde{A}}^{2}\leq c_{1}{\left\|v\right\|}_{A}^{2}, & \end{array}$$

hold. Then, for the preconditioner ${Â}_{a}$ defined by ${Â}_{a}^{-1}:=\Pi {\tilde{A}}^{-1}\Pi^{*}$, there holds the spectral estimate

(43) $$\begin{array}{ll} c_{0}^{-1}{\left(v,v\right)}_{A}\leq {\left({Â}_{a}^{-1}Av,v\right)}_{A}\leq c_{1}{\left(v,v\right)}_{A}\;\forall v\in H. & \end{array}$$

The term auxiliary space method, as coined in Reference 16, refers to the case where the titular fictitious space $\tilde{H}$ is a product space that contains H itself as a component, $\tilde{H}=H\times V_{1}\times \ldots \times V_{n}$, so Π takes the form $\Pi =(I|\Pi_{1}|\ldots |\Pi_{n})$, and $Ã:=\text{diag}(M,{Ā}_{1},\ldots ,{Ā}_{n})$ is a diagonal operator with $M:H\to H^{*}$ and ${Ā}_{j}:V_{j}\to V_{j}^{*}$ and induced norm $\|(v,v_{1},\ldots ,v_{n}){\|}_{Ã}^{2}=\|v{\|}_{M}^{2}+\sum_{j=1}^{n}\|v_{j}{\|}_{{Ā}_{j}}^{2}$. The stability condition (42) then demands the existence of a stable decomposition $v=v_{0}+\sum_{j=1}^{n}v_{j}$ with $v_{0}\in H$ and $v_{j}$ in the range of $\Pi_{j}$. The underlying idea is that the remainder $v_{0}\in H$ in this composition is small and somehow localized and M can be a computationally cheap method (or “smoother”). Often, M is given by some form of additive or multiplicative Schwarz method such as (Block‐)Jacobi or (Block‐)Gauss‐Seidel. In the only relevant case here, where all involved spaces are finite dimensional and n=1, the ASP ${Â}_{a}$ is just

$$\begin{array}{ll} {Â}_{a}^{-1}=\Pi {\tilde{A}}^{-1}\Pi^{*}=\Pi \left(\begin{array}{cc} M^{-1} & 0\\ 0 & {Ā}_{1}^{-1} \end{array}\right)\Pi^{*}=M^{-1}+\Pi_{1}{Ã}_{1}^{-1}\Pi_{1}^{*}. & \end{array}$$

As alluded to by the subscript, ${Â}_{a}$ is an additive preconditioner in that, given some right hand side vector b and intermediate approximation $x^{0}$ with residual $r^{0}:=b-Ax^{0}$, one Richardson iteration with preconditioner ${Â}_{a}$ is to perform

$$\begin{array}{ll} x^{0}\to x^{0}+M^{-1}r^{0}+\Pi_{1}{Ã}_{1}^{-1}\Pi_{1}^{*}r^{0}, & \end{array}$$

that is, to perform two updates additively. The multiplicative ASP ${Â}_{m}$ is implicitly defined by performing these updates successively instead,

$$\begin{array}{llll} x^{1}:=x^{0}+M^{-1}r^{0}, & \;\;r^{1}:=b-Ax^{1},\; & & \\ x^{2}:=x^{1}+\Pi_{1}{Ã}_{1}^{-1}\Pi_{1}^{*}r^{1}, & \;\;r^{2}:=b-Ax^{2},\; & & \end{array}$$

and then, performing another smoothing step with the adjoint smoother $M^{*}$

$$\begin{array}{ll} x^{3}:=x^{2}+{\left(M^{*}\right)}^{-1}r^{2} & \end{array}$$

yielding $x^{3}:=x^{0}+{Â}_{m}^{-1}r^{0}$. Multiplication with ${Â}_{m}^{-1}$ is just performing this procedure once starting with $x^{0}=0$. With symmetry ensured by the additional smoothing step, positive definiteness of ${Â}_{m}$ follows from A≤M and $\Pi_{1}{Ã}_{1}^{-1}\Pi_{1}^{*}\leq A^{-1}$ which can always be achieved by scaling the component preconditioners. If M is a (Block‐)Jacobi preconditioner, scaling of M can be avoided by replacing it with the corresponding (Block‐)Gauss‐Seidel iteration which never over‐corrects, see Reference 33.

Lemma 7 Let an ASP ${Â}_{a}$ with n=1 fulfill the conditions of Lemma 6, and M be either self‐adjoint and positive definite with M≤A or given by (Block‐)Gauss‐Seidel iterations. Let ${Ã}_{1}$ self‐adjoint and positive definite with

(44) $$\begin{array}{ll} \Pi_{1}^{*}A\Pi_{1}\leq {Ã}_{1}. & \end{array}$$

Then ${Â}_{m}$ is self‐adjoint and positive definite and there holds

(45) $$\begin{array}{ll} c_{1}^{-1}{Â}_{m}≲A\leq {Â}_{m}. & \end{array}$$

This is proven within the framework of space decomposition and subspace correction, see Reference 33. The analysis there rests on a strengthened Cauchy‐Schwarz type inequality and a stable decomposition. The former is implied by limited overlap of subspaces and the additional requirements posed on M and ${Ã}_{1}$ and the latter is directly related to (42). See also the discussion in Reference 14 [section 6], where convergence bounds for multiplicative two‐grid Algebraic Multigrid methods are derived from the fictitious space lemma.

## 6 PRECONDITIONERS FOR A

From the point of view of Section 5.2, a straightforward approach to preconditioning A is to use the conforming low order space ${\bar{V}}_{h}$, where preconditioning is well understood and efficient and scalable software is widely available, as basis for an ASP. A slight complication in the analysis arises due to the non‐conformity in boundary conditions between ${𝒱}_{h}=V_{h}\times {\hat{V}}_{h}$, where tangential Dirichlet conditions on $\Gamma_{Ñ}$ are imposed in ${\hat{V}}_{h}$, and ${\bar{V}}_{h}$, where $\Gamma_{Ñ}$ does not feature any Dirichlet conditions. While imposing strong tangential Dirichlet conditions in ${\bar{V}}_{h}$ would sidestep the issue and be convenient for theory, in practice this is only a simple matter when the outflow lies in an axis‐aligned plane and we can impose Dirichlet conditions in the x,y or z component. Therefore, we for now assume that $\Gamma_{Ñ}=\emptyset$ and address the case $\Gamma_{Ñ}\neq \emptyset$ separately in Lemma 9 at the end of this Section.

On ${\bar{V}}_{h}$, we define the bilinear form ā(·,·) (as usual, with associated operator Ā and Finite Element matrix $\bar{A}$) by

(46) $$\begin{array}{ll} ā({ū}_{h},{\bar{v}}_{h}):=\nu^{-1}\int_{\Omega }\varepsilon ({ū}_{h}):\varepsilon ({\bar{v}}_{h})\;\forall {ū}_{h},{\bar{v}}_{h}\in {\bar{V}}_{h}. & \end{array}$$

To define the operator Π in (41) we need the embedding operator

(47) $$\begin{array}{ll} E:{\bar{V}}_{h}\to {𝒱}_{h}:{ū}_{h}\mapsto ({ū}_{h},{\left({ū}_{h}\right)}_{t}) & \end{array}$$

with associated Finite Element matrix E.

Corollary 5 For ${ū}_{h}\in {\bar{V}}_{h}$ there holds

(48) $$\begin{array}{ll} \|E{ū}_{h}{\|}_{A}=\|{ū}_{h}{\|}_{Ā}. & \end{array}$$

For ${ū}_{h}\in {\bar{V}}_{h}$ and $\Gamma_{Ñ}=\emptyset$ there holds $E{ū}_{h}\in {𝒱}_{h}^{c}$ from Lemma 5 and (48) follows from (29).

To establish the stable decomposition (42) we use ℐ from Lemma 2 and define

(49) $$\begin{array}{ll} {ℐ}_{{\bar{V}}_{h}}:{𝒱}_{h}\to {\bar{V}}_{h}:(u_{h},{û}_{h})\mapsto ℐu_{h}. & \end{array}$$

Corollary 6 For $(u_{h},{û}_{h})\in {𝒱}_{h}$ and $\left(w_{h},{ŵ}_{h}):=(I-E{ℐ}_{{\bar{V}}_{h}})(u_{h},{û}_{h}\right)$ there holds

(50) $$\begin{array}{ll} \underset{T\in {𝒯}_{h}}{\sum }(\|\varepsilon (w_{h}){\|}_{T}^{2}+h^{-2}\|w_{h}{\|}_{T}^{2}+\underset{F\in {ℱ}_{T}}{\sum }\|\Pi^{k-1}{\left(w_{h}-{ŵ}_{h}\right)}_{t}{\|}_{j,F}^{2})≲\|(u_{h},{û}_{h}){\|}_{\varepsilon ,h}^{2}, & \end{array}$$

Per definition of E there holds $\Pi^{k-1}{\left(w_{h}-{ŵ}_{h}\right)}_{t}=\Pi^{k-1}{\left(u_{h}-{û}_{h}\right)}_{t}$ and the facet terms are bounded trivially. The volume terms are bounded with Lemma 2 and the identity for the jump terms in (9).

With ${\bar{V}}_{h}$ being of low order, robustness in the polynomial degree k has to be achieved by the smoother.

Theorem 2 Let M be the overlapping Block‐Jacobi preconditioner for A that has one block per facet $F\in {ℱ}_{h}$ that contains all ${𝒱}_{h}$ degrees of freedom associated to either F or any $T\in {𝒯}_{h}$ such that $F\in {ℱ}_{T}$. Let C be an SPD preconditioner for $\bar{A}$ such that $C∼\bar{A}$. Then, conditions (41) and (42) of Lemma 6 are fulfilled for $H={𝒱}_{h}$, $\tilde{H}={𝒱}_{h}\times {\bar{V}}_{h}$,

$$\begin{array}{ll} \Pi :\tilde{H}\to H:((u_{h},{û}_{h}),{ū}_{h})\mapsto (u_{h},{û}_{h})+E{ū}_{h}, & \end{array}$$

and Ã:=diag(M,C) with $c_{0}≲1$ and $c_{1}≲\gamma \cdot {\left(\log k\right)}^{3}$. That is, for ${\hat{A}}_{a}^{-1}:=M^{-1}+EC^{-1}E^{T}$ there holds

$$\begin{array}{ll} {\hat{A}}_{a}≲A≲\gamma \cdot {\left(\log k\right)}^{3}{\hat{A}}_{a}. & \end{array}$$

We postpone the proof of Theorem 2 to Section 6.1, where we discuss preconditioning of $A^{\partial }$, as obtaining the logarithmic bound in k is more natural in that context.

Remark 5 If one is satisfied with a polynomial bound in k, Theorem 2 can be shown only using standard Finite Element inverse estimates and Corollaries 5 and 6.

### 6.1 Preconditioning via the condensed system

Using the factorization in (32) to implement multiplication with A also opens up a way to precondition it. Replacing $A^{\partial }$ by some preconditioner ${\hat{A}}^{\partial }$ yields

$$\begin{array}{ll} {\hat{A}}^{\text{ext}}:=\left(\begin{array}{cc} I & A_{\partial \circ }A_{\circ \circ }^{-1}\\ 0 & I \end{array}\right)\left(\begin{array}{cc} {\hat{A}}^{\partial } & 0\\ 0 & A_{\circ \circ } \end{array}\right)\left(\begin{array}{cc} I & 0\\ A_{\circ \circ }^{-1}A_{\circ \partial } & I \end{array}\right) & \end{array}$$

as a preconditioner for A. From the factorization (32) it clearly follows that

$$\begin{array}{ll} c_{1}{\hat{A}}^{\text{ext}}\leq A\leq c_{2}{\hat{A}}^{\text{ext}}\;\Leftrightarrow \;c_{1}{\hat{A}}^{\partial }\leq A^{\partial }\leq c_{2}{\hat{A}}^{\partial } & \end{array}$$

and we are left with the task to precondition the “double” Schur complement $A^{\partial }$. Analogues for $A^{\partial }$ of the preconditioners ${\hat{A}}_{a}$ and ${\hat{A}}_{m}$ can be constructed straightforwardly with the modified embedding operator

(51) $$\begin{array}{ll} E^{\partial }:{\bar{V}}_{h}\to {𝒱}_{h}^{\text{harm}}:{ū}_{h}\mapsto ℋE{ū}_{h}. & \end{array}$$

Note that the matrix $E^{\partial }{∼}_{G}^{\partial }\;E^{\partial }$ is just a sub‐matrix of E as ℋ does not change $\left(u^{\partial },û\right)$ coefficients, see (34), with

$$\begin{array}{ll} E=\left(\begin{array}{c} E_{\circ }\\ E_{\partial }\\ E_{û} \end{array}\right)\;\text{we have}\;E^{\partial }=\left(\begin{array}{c} E_{\partial }\\ E_{û} \end{array}\right). & \end{array}$$

We could modify the bilinear form in ${\bar{V}}_{h}$ and use $\bar{A}:=E^{\partial ,T}A^{\partial }E^{\partial }$, which would be computable element‐wise. In that case, the exact analogue of Corollary 5 would hold. However, as we now show, this is not strictly necessary and for ease of implementation we opt to keep $\bar{A}$ defined by (46).

Lemma 8 For $(u_{h},{û}_{h})\in {𝒱}_{h}$ there holds

(52) $$\begin{array}{ll} ℐu_{h}^{\circ } & =0, \end{array}$$

(53) $$\begin{array}{ll} {ℐ}_{{\bar{V}}_{h}}(u_{h},{û}_{h}) & ={ℐ}_{{\bar{V}}_{h}}ℋ(u_{h},{û}_{h})={ℐ}_{{\bar{V}}_{h}}{ℋ}_{\varepsilon }(u_{h},{û}_{h}). \end{array}$$

Any $u_{h}^{\circ }\in V_{h}^{\circ }$ restricted to $T\in {𝒯}_{h}$ is a normal bubble. At any vertex p of T, d linearly independent components of ${\left(u_{h}^{\circ }\right)}_{|T}(p)$ vanish, and therefore ${\left(u_{h}^{\circ }\right)}_{|T}(p)$ and $ℐu_{h}^{\circ }$ also vanish as a whole. This concludes the proof as $ℋ,{ℋ}_{\varepsilon }$ only add some $v_{h}\in V_{h}^{\circ }$ to the $V_{h}$ component of $(u_{h},{û}_{h})\in {𝒱}_{h}$.

Corollary 7 For ${ū}_{h}\in {\bar{V}}_{h}$ there holds

(54) $$\begin{array}{ll} \gamma^{-1}\|{ū}_{h}{\|}_{Ā}≲\gamma^{-1}\nu \|{ℋ}_{\varepsilon }E{ū}_{h}{\|}_{\varepsilon ,h,\partial }≲\|E^{\partial }{ū}_{h}{\|}_{A^{\partial }}\leq \|{ū}_{h}{\|}_{Ā}. & \end{array}$$

The sharp upper bound is a consequence of the energy minimization (33) and Corollary 5,

$$\begin{array}{ll} \|E^{\partial }{ū}_{h}{\|}_{A^{\partial }}^{2}=\|ℋE{ū}_{h}{\|}_{A}^{2}\leq \|E{ū}_{h}{\|}_{A}^{2}=\|{ū}_{h}{\|}_{Ā}^{2}. & \end{array}$$

With $(u_{h},{û}_{h}):={ℋ}_{\varepsilon }E{ū}_{h}$ and (52) we conclude the identity $ℐu_{h}=ℐ{ℋ}_{\varepsilon }E{ū}_{h}=ℐE{ū}_{h}={ū}_{h}$. Now, (17) and (9) show

$$\begin{array}{llll} \|{ū}_{h}{\|}_{Ā}^{2}=\nu \|\varepsilon (ℐu_{h}){\|}_{0}^{2} & ≲\nu \|\varepsilon (u_{h}){\|}_{0}^{2}+\nu \|\varepsilon (ℐu_{h}-u_{h}){\|}_{0}^{2}≲\nu \|(u_{h},{û}_{h}){\|}_{\varepsilon ,h}^{2}=\nu \|{ℋ}_{\varepsilon }E{ū}_{h}{\|}_{\varepsilon ,h}^{2}\; & & \end{array}$$

and the rest follows form the lower bound in (38).

Corollary 8 For $(u_{h},{û}_{h})\in {𝒱}_{h}$ and either $\left(w_{h},{ŵ}_{h}):={ℋ}_{\varepsilon }(I-E{ℐ}_{{\bar{V}}_{h}})(u_{h},{û}_{h}\right)$ or $\left(w_{h},{ŵ}_{h}):=ℋ(I-E{ℐ}_{{\bar{V}}_{h}})(u_{h},{û}_{h}\right)$ there holds

(55) $$\begin{array}{ll} \underset{T\in {𝒯}_{h}}{\sum }(\|\nabla w_{h}{\|}_{T}^{2}+h^{-2}\|w_{h}{\|}_{T}^{2}+\underset{F\in {ℱ}_{T}}{\sum }\|\Pi^{k-1}{\left(w_{h}-{ŵ}_{h}\right)}_{t}{\|}_{j,F}^{2})≲\|(u_{h},{û}_{h}){\|}_{\varepsilon ,h,\partial }^{2}. & \end{array}$$

With the readily apparent ${ℐ}_{{\bar{V}}_{h}}E{ℐ}_{{\bar{V}}_{h}}={ℐ}_{{\bar{V}}_{h}}$ and (53) we see

$$\begin{array}{ll} E{ℐ}_{{\bar{V}}_{h}}{ℋ}_{\varepsilon }(I-E{ℐ}_{{\bar{V}}_{h}})=0\;\text{and}\;{ℋ}_{\varepsilon }(I-E{ℐ}_{{\bar{V}}_{h}})(u_{h},{û}_{h})={ℋ}_{\varepsilon }(I-E{ℐ}_{{\bar{V}}_{h}}){ℋ}_{\varepsilon }(u_{h},{û}_{h}). & \end{array}$$

This lets us insert a zero into $\left(w_{h},{ŵ}_{h}\right)$ to obtain an expression without ${ℋ}_{\varepsilon }$ in front,

$$\begin{array}{ll} (w_{h},{ŵ}_{h})={ℋ}_{\varepsilon }(I-E{ℐ}_{{\bar{V}}_{h}})(u_{h},{û}_{h})=(I-E{ℐ}_{{\bar{V}}_{h}}){ℋ}_{\varepsilon }(I-E{ℐ}_{{\bar{V}}_{h}}){ℋ}_{\varepsilon }(u_{h},{û}_{h}). & \end{array}$$

Corollary 6 applied to ${ℋ}_{\varepsilon }(I-E^{\partial }{ℐ}_{{\bar{V}}_{h}}){ℋ}_{\varepsilon }(u_{h},{û}_{h})$ shows

$$\begin{array}{llll} \underset{T\in {𝒯}_{h}}{\sum }(h^{-2}\|w_{h}{\|}_{0}^{2}+ & \|\nabla w_{h}{\|}_{0}^{2}+\underset{F\in {ℱ}_{T}}{\sum }h^{-1}\|\Pi^{k-1}{\left(w_{h}-{ŵ}_{h}\right)}_{t}{\|}_{j,F}^{2})≲\|{ℋ}_{\varepsilon }(I-E{ℐ}_{{\bar{V}}_{h}}){ℋ}_{\varepsilon }(u_{h},{û}_{h}){\|}_{\varepsilon ,h}^{2}.\; & & \end{array}$$

The proof is concluded by the energy minimization of ${ℋ}_{\varepsilon }$,

$$\begin{array}{llll} \|{ℋ}_{\varepsilon }(I-E{ℐ}_{{\bar{V}}_{h}}){ℋ}_{\varepsilon }(u_{h},{û}_{h}){\|}_{\varepsilon ,h}^{2} & \leq \|(I-E{ℐ}_{{\bar{V}}_{h}}){ℋ}_{\varepsilon }(u_{h},{û}_{h}){\|}_{\varepsilon ,h}^{2}≲\|{ℋ}_{\varepsilon }(u_{h},{û}_{h}){\|}_{\varepsilon ,h}^{2}=\|(u_{h},{û}_{h}){\|}_{\varepsilon ,h,\partial }^{2},\; & & \end{array}$$

where the continuity of $E{ℐ}_{{\bar{V}}_{h}}$ in the $\|\cdot {\|}_{\varepsilon ,h}$ norm follows from Lemma 2. The other case $\left(w_{h},{ŵ}_{h}):=ℋ(I-E{ℐ}_{{\bar{V}}_{h}})(u_{h},{û}_{h}\right)$ works analogously.

An operator that, like $E{ℐ}_{{\bar{V}}_{h}}$, extracts a low order component out of $(u_{h},{û}_{h})\in {𝒱}_{h}$ is

(56) $$\begin{array}{ll} \Pi^{\mathrm{lo}}:=\left\{\begin{array}{c} {𝒱}_{h}\to (V_{h}\cap {\mathbb{P}}^{1}({𝒯}_{h},{\mathbb{R}}^{d}))\times ({\hat{V}}_{h}\cap {\mathbb{P}}^{1}({ℱ}_{h},{\mathbb{R}}^{d}))\\ (u_{h},{û}_{h})\mapsto ({ℐ}_{\text{BDM}}^{1}u_{h},\Pi_{F}^{1}{û}_{h}), \end{array}\right. & \end{array}$$

where ${ℐ}_{\text{BDM}}^{1}$ is the standard ${\text{BDM}}^{1}$ interpolator, see Reference 19, that is, $\forall F\in {ℱ}_{h}$ there holds ${\left({ℐ}_{\text{BDM}}^{1}u_{h}\right)}_{n}={\left(\Pi_{F}^{1}(u_{h})\right)}_{n}$.

Corollary 9 For $(u_{h},{û}_{h})\in {𝒱}_{h}^{\text{harm}}$ and $\left(w_{h},{ŵ}_{h}):={ℋ}_{\varepsilon }(I-\Pi^{\mathrm{lo}})(u_{h},{û}_{h}\right)$ there holds

(57) $$\begin{array}{ll} \underset{T\in {𝒯}_{h}}{\sum }(\|\nabla w_{h}{\|}_{T}^{2}+h^{-2}\|w_{h}{\|}_{T}^{2}+\underset{F\in {ℱ}_{T}}{\sum }\|\Pi^{k-1}{\left(w_{h}-{ŵ}_{h}\right)}_{t}{\|}_{j,F}^{2})≲\|(u_{h},{û}_{h}){\|}_{\varepsilon ,h,\partial }^{2}. & \end{array}$$

Follows from the Bramble‐Hilbert Lemma, an element‐level Korn inequality and Lemma 8 with similar arguments as the previous Corollary 8.

Theorem 3 Let $M^{\partial }$ be the block Jacobi preconditioner for $A^{\partial }$, consisting of one block per facet $F\in {ℱ}_{h}$ that contains all ${𝒱}_{h}^{\text{harm}}$ degrees of freedom associated to F. Let C be an SPD preconditioner for $\bar{A}$ such that $C∼\bar{A}$. Then, conditions (41) and (42) of Lemma 6 are fulfilled for $H={𝒱}_{h}^{\text{harm}}$, $\tilde{H}={𝒱}_{h}^{\text{harm}}\times {\bar{V}}_{h}$,

$$\begin{array}{ll} \Pi :\tilde{H}\to H:((u_{h},{û}_{h}),{ū}_{h})\mapsto (u_{h},{û}_{h})+E^{\partial }{ū}_{h}, & \end{array}$$

and $Ã:=\mathrm{diag}(M^{\partial },C)$ with $c_{0}≲1$ and $c_{1}≲\gamma \cdot {\left(\log k\right)}^{3}$. That is, for ${\left({\hat{A}}_{a}^{\partial }\right)}^{-1}:={\left(M^{\partial }\right)}^{-1}+E^{\partial }C^{-1}E^{\partial ,T}$ there holds

$$\begin{array}{ll} {\hat{A}}_{a}^{\partial }≲A^{\partial }≲\gamma \cdot {\left(\log k\right)}^{3}{\hat{A}}_{a}^{\partial }. & \end{array}$$

The continuity condition (41) holds as $\|E^{\partial }{ū}_{h}{\|}_{A^{\partial }}\leq \|{ū}_{h}{\|}_{Ā}$ is shown in Corollary 7 and $\|(u_{h},{û}_{h}){\|}_{A^{\partial }}^{2}≲\|(u_{h},{û}_{h}){\|}_{M^{\partial }}^{2}$ follows from limited overlap of basis functions. For some $(u_{h},{û}_{h})\in H={𝒱}_{h}$, the choice

$$\begin{array}{ll} \tilde{v}:=((I-E^{\partial }{ℐ}_{{\bar{V}}_{h}})(u_{h},{û}_{h}),{ℐ}_{{\bar{V}}_{h}}(u_{h},{û}_{h}))\in \tilde{H}, & \end{array}$$

fulfills $(u_{h},{û}_{h})=\Pi \tilde{v}$. The stability condition (42) is verified by showing

(58) $$\begin{array}{ll} \|(I-E^{\partial }{ℐ}_{{\bar{V}}_{h}})(u_{h},{û}_{h}){\|}_{M^{\partial }}^{2}+\|{ℐ}_{{\bar{V}}_{h}}(u_{h},{û}_{h}){\|}_{C}^{2}≲\gamma \cdot {\left(\log k\right)}^{3}\|(u_{h},{û}_{h}){\|}_{A^{\partial }}^{2}. & \end{array}$$

For the second term, C≲Ā and Corollary 7 bound it by $\|E^{\partial }{ℐ}_{{\bar{V}}_{h}}(u_{h},{û}_{h}){\|}_{\varepsilon ,h,\partial }^{2}$ which is then further bounded by the $\|\cdot {\|}_{A^{\partial }}$ norm with the continuity of $E^{\partial }{ℐ}_{{\bar{V}}_{h}}$ in $\|\cdot {\|}_{\varepsilon ,h,\partial }$, as implied by Corollary 8, and (38) where we incur the factor γ. The other bound requires a more careful approach. For general $(v_{h},{\hat{v}}_{h})\in {𝒱}_{h}^{\text{harm}}$, and therefore also for $(I-E^{\partial }{ℐ}_{{\bar{V}}_{h}})(u_{h},{û}_{h})\in {𝒱}_{h}^{\text{harm}}$, the lower bound in (38) shows

(59) $$\begin{array}{ll} \nu \underset{F\in {ℱ}_{h}}{\sum }\|(v_{h},{\hat{v}}_{h}){\|}_{\varepsilon ,F}^{2}≲\nu \|(v_{h},{\hat{v}}_{h}){\|}_{\varepsilon ,h,\partial }^{2}≲\gamma \|(v_{h},{\hat{v}}_{h}){\|}_{A^{\partial }}^{2}. & \end{array}$$

In the first step we bounded the facet terms in the sum, where an infimum is taken over functions with arbitrary traces on neighboring faces, by $\|(v_{h},{\hat{v}}_{h}){\|}_{\varepsilon ,h,\partial }$, where these traces are fixed. On the other hand, the upper inequality in (38) shows

(60) $$\begin{array}{ll} \|(v_{h},{\hat{v}}_{h}){\|}_{M^{\partial }}^{2}=\underset{F\in {ℱ}_{h}}{\sum }\|{\left(v_{h},{\hat{v}}_{h}\right)}^{\left(F\right)}{\|}_{A^{\partial }}^{2}≲\nu \underset{F\in {ℱ}_{h}}{\sum }\|(v_{h},{\hat{v}}_{h}){\|}_{\varepsilon ,F,0}^{2}, & \end{array}$$

where ${\left(v_{h},{\hat{v}}_{h}\right)}^{\left(F\right)}$ denotes the element of ${𝒱}_{h}^{\text{harm}}$ that has the same coordinates as $\left(v_{h},{\hat{v}}_{h}\right)$ for degrees of freedom associated to F and whose degrees of freedom are zero otherwise (Galerkin isomorphism ${∼}_{G}^{\partial }$). Given the continuity of $E^{\partial }{ℐ}_{{\bar{V}}_{h}}$ in $\|\cdot {\|}_{\varepsilon ,h,\partial }$ (see Corollary 8), the crucial step is therefore to bound $\|\cdot {\|}_{\varepsilon ,F,0}$ by $\|\cdot {\|}_{\varepsilon ,F}$, as in Corollary 1. However, Corollary 1 is only applicable if $\Pi_{F}^{R}({\left(v_{h}\right)}_{n}n+{\hat{v}}_{h})=0\;\forall F\in {ℱ}_{h}$, which is not usually true for $\left(I-E^{\partial }{ℐ}_{{\bar{V}}_{h}})(u_{h},{û}_{h}\right)$.

This does not pose a problem for low‐order functions or, crucially, their harmonic extensions, where an alternative path via an inverse inequality bypasses the trace estimate. For a low order $\left(v_{h},{\hat{v}}_{h})\in {𝒱}_{h}\cap ({\mathbb{P}}^{1}({𝒯}_{h})\times {\mathbb{P}}^{1}({ℱ}_{h})\right)$, a standard, and necessarily k‐independent, inverse estimate is

(61) $$\begin{array}{ll} \underset{F\in {ℱ}_{h}}{\sum }\|{\left(v_{h},{\hat{v}}_{h}\right)}^{\left(F\right)}{\|}_{\varepsilon ,h}^{2}≲\underset{T\in {𝒯}_{h}}{\sum }h^{-2}\|v_{h}{\|}_{T}^{2}+\underset{F\in {ℱ}_{h}}{\sum }\|\Pi^{k-1}{\left(v_{h}-{\hat{v}}_{h}\right)}_{t}{\|}_{j,F}^{2}. & \end{array}$$

Because of the energy minimization in $\|\cdot {\|}_{\varepsilon ,h,\partial }$, the estimate holds with the same constant also for discrete harmonic extensions $\left(v_{h},{\hat{v}}_{h})\in {ℋ}_{\varepsilon }({𝒱}_{h}\cap ({\mathbb{P}}^{1}({𝒯}_{h})\times {\mathbb{P}}^{1}({ℱ}_{h}))\right)$ of these low order functions where $\|{\left(v_{h},{\hat{v}}_{h}\right)}^{\left(F\right)}{\|}_{\varepsilon ,h}=\|{\left(v_{h},{\hat{v}}_{h}\right)}^{\left(F\right)}{\|}_{\varepsilon ,h,\partial }$. The right hand side can then further be bounded using Corollary 8 or Corollary 9 if $\left(v_{h},{\hat{v}}_{h}\right)$ takes the form of an approximation error, as required there.

Therefore, the strategy is to use the operator $\Pi^{\mathrm{lo}}$ as defined in (56) to split the $\|\cdot {\|}_{M^{\partial }}$ term in (58) into low and high order components. The former can then be bounded via the inverse estimate and the latter via the trace inequality, we have

$$\begin{array}{llll} \|(I-E^{\partial }{ℐ}_{{\bar{V}}_{h}})(u_{h},{û}_{h}){\|}_{M^{\partial }}^{2}\;≲\; & \|ℋ\Pi^{\mathrm{lo}}(I-E^{\partial }{ℐ}_{{\bar{V}}_{h}})(u_{h},{û}_{h}){\|}_{M^{\partial }}^{2}+\|(I-ℋ\Pi^{\mathrm{lo}})(I-E^{\partial }{ℐ}_{{\bar{V}}_{h}})(u_{h},{û}_{h}){\|}_{M^{\partial }}^{2}.\; & & \end{array}$$

For $(v_{h},{\hat{v}}_{h})\in {𝒱}_{h}$, the (low order) normal trace of the $V_{h}$ component of $E{ℐ}_{{\bar{V}}_{h}}(v_{h},{\hat{v}}_{h})$ and the entire ${\hat{V}}_{h}$ component are not changed by ℋ and there holds

$$\begin{array}{ll} \Pi^{\mathrm{lo}}E^{\partial }{ℐ}_{{\bar{V}}_{h}}(v_{h},{\hat{v}}_{h})=\Pi^{\mathrm{lo}}ℋE{ℐ}_{{\bar{V}}_{h}}(v_{h},{\hat{v}}_{h})=\Pi^{\mathrm{lo}}E{ℐ}_{{\bar{V}}_{h}}(v_{h},{\hat{v}}_{h})=E{ℐ}_{{\bar{V}}_{h}}(v_{h},{\hat{v}}_{h}), & \end{array}$$

that is $\Pi^{\mathrm{lo}}E^{\partial }{ℐ}_{{\bar{V}}_{h}}=E{ℐ}_{{\bar{V}}_{h}}$. Therefore $ℋ\Pi^{\mathrm{lo}}E^{\partial }{ℐ}_{{\bar{V}}_{h}}=ℋE{ℐ}_{{\bar{V}}_{h}}=E^{\partial }{ℐ}_{{\bar{V}}_{h}}$ and the high order term can be simplified,

$$\begin{array}{llll} \|(I-ℋ\Pi^{\mathrm{lo}})(I-E^{\partial }{ℐ}_{{\bar{V}}_{h}})(u_{h},{û}_{h}){\|}_{M^{\partial }}^{2}= & \|(I-ℋ\Pi^{\mathrm{lo}})(u_{h},{û}_{h}){\|}_{M^{\partial }}^{2}.\; & & \end{array}$$

Note that we can apply Corollary 1 not only to $\left(I-\Pi^{\mathrm{lo}})(u_{h},{û}_{h}\right)$, which is apparent from the definition of $\Pi^{\mathrm{lo}}$, but also to $\left(I-ℋ\Pi^{\mathrm{lo}})(u_{h},{û}_{h}\right)$ because again, as argued above, ℋ does not change the relevant traces. Therefore, (60), (21) and then (59) shows

$$\begin{array}{llll} \|(I-ℋ\Pi^{\mathrm{lo}})(u_{h},{û}_{h}){\|}_{M^{\partial }}^{2}≲ & \nu {\left(\log k\right)}^{3}\|(I-ℋ\Pi^{\mathrm{lo}})(u_{h},{û}_{h}){\|}_{\varepsilon ,h,\partial }^{2}≲\nu {\left(\log k\right)}^{3}\|(u_{h},{û}_{h}){\|}_{\varepsilon ,h,\partial }^{2},\; & & \end{array}$$

where the continuity of $ℋ\Pi^{\mathrm{lo}}$ used in the last estimate follows from the Bramble Hilbert Lemma as in the proof of Corollary 9. Finally, the bound

$$\begin{array}{llll} \|(I-ℋ\Pi^{\mathrm{lo}})(I-E^{\partial }{ℐ}_{{\bar{V}}_{h}})(u_{h},{û}_{h}){\|}_{M^{\partial }}^{2}≲ & \gamma \cdot {\left(\log k\right)}^{3}\|(u_{h},{û}_{h}){\|}_{A^{\partial }}^{2},\; & & \end{array}$$

follows with (38). As for the low order term, with $ℋ\Pi^{\mathrm{lo}}E^{\partial }{ℐ}_{{\bar{V}}_{h}}=ℋE{ℐ}_{{\bar{V}}_{h}}$ we see

$$\begin{array}{llll} \|ℋ\Pi^{\mathrm{lo}}(I-E^{\partial }{ℐ}_{{\bar{V}}_{h}})(u_{h},{û}_{h}){\|}_{M^{\partial }}^{2} & =\|ℋ(\Pi^{\mathrm{lo}}-E{ℐ}_{{\bar{V}}_{h}})(u_{h},{û}_{h}){\|}_{M^{\partial }}^{2},\; & & \end{array}$$

and applying (61) to the (harmonic extension of) the low order function $\left(\Pi^{\mathrm{lo}}-E{ℐ}_{{\bar{V}}_{h}})(u_{h},{û}_{h}\right)$ gives

$$\begin{array}{llll} \|ℋ\Pi^{\mathrm{lo}}(I-E^{\partial }{ℐ}_{{\bar{V}}_{h}})(u_{h},{û}_{h}){\|}_{M^{\partial }}^{2}\leq \nu & \underset{F\in {ℱ}_{h}}{\sum }\|{\left(ℋ(\Pi^{\mathrm{lo}}-E{ℐ}_{{\bar{V}}_{h}})(u_{h},{û}_{h})\right)}^{\left(F\right)}{\|}_{\varepsilon ,h,\partial }^{2}\; & & \\ =\nu & \underset{F\in {ℱ}_{h}}{\sum }\|{\left({ℋ}_{\varepsilon }(\Pi^{\mathrm{lo}}-E{ℐ}_{{\bar{V}}_{h}})(u_{h},{û}_{h})\right)}^{\left(F\right)}{\|}_{\varepsilon ,h}^{2}\; & & \\ ≲\nu & (\underset{T\in {𝒯}_{h}}{\sum }h^{-2}\|w_{h}{\|}_{T}^{2}+\underset{F\in {ℱ}_{T}}{\sum }\|\Pi^{k-1}{\left(w_{h}-{ŵ}_{h}\right)}_{t}{\|}_{j,F}^{2}),\; & & \end{array}$$

where we write $\left(w_{h},{ŵ}_{h}):={ℋ}_{\varepsilon }(\Pi^{\mathrm{lo}}-E{ℐ}_{{\bar{V}}_{h}})(u_{h},{û}_{h}\right)$. We further split $\left(w_{h},{ŵ}_{h}\right)$ into $\left(\alpha_{h},{\hat{\alpha }}_{h}):={ℋ}_{\varepsilon }(I-E{ℐ}_{{\bar{V}}_{h}})(u_{h},{û}_{h}\right)$ and $\left(\beta_{h},{\hat{\beta }}_{h}):={ℋ}_{\varepsilon }(I-\Pi^{\mathrm{lo}})(u_{h},{û}_{h}\right)$ and get

$$\begin{array}{llll} \|ℋ\Pi^{\mathrm{lo}}(I-E^{\partial }{ℐ}_{{\bar{V}}_{h}})(u_{h},{û}_{h}){\|}_{M^{\partial }}^{2}≲ & \nu (\underset{T\in {𝒯}_{h}}{\sum }h^{-2}\|\alpha_{h}{\|}_{T}^{2}+\underset{F\in {ℱ}_{T}}{\sum }\|\Pi^{k-1}{\left(\alpha_{h}-{\hat{\alpha }}_{h}\right)}_{t}{\|}_{j,F}^{2})\; & & \\ & +\nu (\underset{T\in {𝒯}_{h}}{\sum }h^{-2}\|\beta_{h}{\|}_{T}^{2}+\underset{F\in {ℱ}_{T}}{\sum }\|\Pi^{k-1}{\left(\beta_{h}-{\hat{\beta }}_{h}\right)}_{t}{\|}_{j,F}^{2}).\; & & \end{array}$$

Corollary 8 and (38) bound the former two terms,

$$\begin{array}{ll} \nu (\underset{T\in {𝒯}_{h}}{\sum }h^{-2}\|\alpha_{h}{\|}_{T}^{2}+\underset{F\in {ℱ}_{T}}{\sum }\|\Pi^{k-1}{\left(\alpha_{h}-{\hat{\alpha }}_{h}\right)}_{t}{\|}_{j,F}^{2})≲\nu \|(u_{h},{û}_{h}){\|}_{\varepsilon ,h,\partial }≲\gamma \|(u_{h},{û}_{h}){\|}_{A^{\partial }}, & \end{array}$$

and Corollary 9 and (38) the latter two,

$$\begin{array}{ll} \nu (h^{-2}\|\beta_{h}{\|}_{T}^{2}+\underset{F\in {ℱ}_{T}}{\sum }\|\Pi^{k-1}{\left(\beta_{h}-{\hat{\beta }}_{h}\right)}_{t}{\|}_{j,F}^{2})≲\nu \|(u_{h},{û}_{h}){\|}_{\varepsilon ,h,\partial }≲\gamma \|(u_{h},{û}_{h}){\|}_{A^{\partial }}. & \end{array}$$

Proof of Theorem 2 Similarly to the proof of Theorem 3, the continuity condition follows from Corollary 5, limited overlap of basis functions and this time also limited overlap of the Jacobi blocks themselves. Also similarly, the stability condition is proven by setting $\tilde{v}:=((I-E{ℐ}_{{\bar{V}}_{h}})(u_{h},{û}_{h}),{ℐ}_{{\bar{V}}_{h}}(u_{h},{û}_{h}))\in \tilde{H}$ and using Corollary 6. The bound $\|(I-E{ℐ}_{{\bar{V}}_{h}})(u_{h},{û}_{h}){\|}_{M}^{2}≲\|(u_{h},{û}_{h}){\|}_{A}^{2}$ via the triangle inequality follows from

$$\begin{array}{ll} \|(I-E^{\partial }{ℐ}_{{\bar{V}}_{h}})(u_{h},{û}_{h}){\|}_{M}^{2}≲\gamma \cdot {\left(\log k\right)}^{3}\|(u_{h},{û}_{h}){\|}_{A}^{2}, & \end{array}$$

which was already shown in the proof of Theorem 3, and the estimate

$$\begin{array}{ll} \|(E-E^{\partial }){ℐ}_{{\bar{V}}_{h}}(u_{h},{û}_{h}){\|}_{M}^{2}=\|(I-ℋ)E{ℐ}_{{\bar{V}}_{h}}(u_{h},{û}_{h}){\|}_{M}^{2}≲\|(u_{h},{û}_{h}){\|}_{A}^{2}. & \end{array}$$

The latter holds because $\left(I-ℋ)E{ℐ}_{{\bar{V}}_{h}}(u_{h},{û}_{h}\right)$ is a normal bubble, that is all its coupling degrees of freedom are zero, and A restricted to such functions is block diagonal.

Corollary 10 Let ${\hat{A}}_{m}$ and ${\hat{A}}_{m}^{\partial }$ be the multiplicative versions of $\hat{A}$ and ${\hat{A}}^{\partial }$, respectively, with the Block‐Jacobi smoothers M, $M^{\partial }$ replaced by Block‐Gauss‐Seidel sweeps and let $\bar{A}\leq C$. Then there holds

(62) $$\begin{array}{ll} \gamma^{-1}\cdot {\left(\log k\right)}^{-3}{\hat{A}}_{m}≲ & \;A\leq {\hat{A}}_{m}, \end{array}$$

(63) $$\begin{array}{ll} \gamma^{-1}\cdot {\left(\log k\right)}^{-3}{\hat{A}}_{m}^{\partial }≲ & \;A^{\partial }\leq {\hat{A}}_{m}^{\partial }. \end{array}$$

The former result (62) follows from Theorem 2 and Lemma 7, where condition (44) is fulfilled due to $\bar{A}\leq C$ and Corollary 5. The latter one (63) follows along the same lines with Theorem 3 and the strict upper bound in (54) for Lemma 7.

Remark 6 Although we have only experimental evidence that the constant γ in Theorems 2 and 3 is benign, the proofs of these theorems show that in the $\|\cdot {\|}_{\varepsilon ,h}$ and $\|\cdot {\|}_{\varepsilon ,h,\partial }$ norm they hold independently of γ. That is, we have results for ASPs for HDG methods with optimal stabilization that are explicit and robust in k.

### 6.2 Non‐conformity in boundary conditions

We now return to the case of $\Gamma_{Ñ}\neq \emptyset$. Instead of enforcing zero tangential Dirichlet conditions on $\Gamma_{Ñ}$ in ${\bar{V}}_{h}$, it suffices to add a tangential penalty to Ā and for E to zero out ${\hat{V}}_{h}$ degrees of freedom on $\Gamma_{Ñ}$.

Lemma 9 For some C>0, let Ā be defined by the modified bilinear form

$$\begin{array}{ll} ā({ū}_{h},{\bar{v}}_{h}):=\int_{\Omega }\nu \varepsilon ({ū}_{h}):\varepsilon ({\bar{v}}_{h})+\underset{F\subseteq \Gamma_{Ñ}}{\sum }\int_{F}\frac{\nu Ck^{2}}{h}{\left({ū}_{h}\right)}_{t}{\left({\bar{v}}_{h}\right)}_{t}. & \end{array}$$

and $\pi_{0}:{𝒱}_{h}\to {𝒱}_{h}$ be the operator that zeros out ${\hat{V}}_{h}$ degrees of freedom on $\Gamma_{Ñ}$. Then, for C large enough there holds

$$\begin{array}{llll} \|{ū}_{h}{\|}_{Ā}^{2} & ≲\|\pi_{0}E{ū}_{h}{\|}_{A}^{2}\leq \|{ū}_{h}{\|}_{Ā}^{2},\;\text{and}\;\|{ū}_{h}{\|}_{Ā}^{2}≲\|ℋ\pi_{0}E{ū}_{h}{\|}_{A^{\partial }}^{2}\leq \|{ū}_{h}{\|}_{Ā}^{2}.\; & & \end{array}$$

These estimates are robust in k.

With the upper bound in (28) and (9) there holds

$$\begin{array}{llll} \|\pi_{0}E{ū}_{h}{\|}_{A}^{2} & \leq \nu (\|\varepsilon ({ū}_{h}){\|}_{0}^{2}+\underset{F\in {ℱ}_{h}^{Ñ}}{\sum }\|\Pi^{k-1}{\left({ū}_{h}\right)}_{t}{\|}_{j,F}^{2})≲\nu (\|\varepsilon ({ū}_{h}){\|}_{0}^{2}+\underset{F\in {ℱ}_{h}^{Ñ}}{\sum }\frac{k^{2}}{h}\|\Pi^{k-1}{\left({ū}_{h}\right)}_{t}{\|}_{F}^{2}),\; & & \end{array}$$

that is for large enough C we have $\|\pi_{0}E{ū}_{h}{\|}_{A}^{2}\leq \|{ū}_{h}{\|}_{Ā}^{2}$. The lower bound $\|{ū}_{h}{\|}_{Ā}^{2}≲\|\pi_{0}E{ū}_{h}{\|}_{A}^{2}$ similarly follows from the lower bound in (28) and the fact that, as ${ū}_{h}\in {\mathbb{P}}^{1}({𝒯}_{h})$ is of low order, the high order terms in (9) vanish and there holds

$$\begin{array}{ll} \frac{k^{2}}{h}\|\Pi^{k-1}{\left({ū}_{h}\right)}_{t}{\|}_{F}^{2}∼\|\Pi^{k-1}{\left({ū}_{h}\right)}_{t}{\|}_{j,F}^{2}, & \end{array}$$

with a k‐robust constant. The estimates for the $\|\cdot {\|}_{A^{\partial }}$‐norm follow from the ones for the $\|\cdot {\|}_{A}$‐norm with energy minimization as in the proof of Corollary 7.

Modifying Ā and the embedding operators like this, one shows the proofs of Section 6 also for the case $\Gamma_{Ñ}\neq \emptyset$.

## 7 THE LOWEST ORDER CASE

The MCS method of Section 4 is, as already mentioned there, not stable in the lowest order case k=1. Stability of the method is recovered when a simplified stress tensor σ=−ν∇(u) is used in (1a), but we are interested in treating the full symmetric stress tensor σ=−νε(u). The five coupling degrees of freedom per facet we have for k=1, that is three in $V_{h}\subseteq {\text{BDM}}^{1}$ and two enforced by ${\hat{V}}_{h}$, are too few to capture the six rigid body modes.

In Reference 9, this was remedied by using a vector‐valued $W_{h}$ instead of the 𝕂‐valued one here, which just means that all occurrences of $\omega_{h}$ have to be replaced by $\kappa (\omega_{h})$ everywhere, and taking it as a subset of H(div),

$$\begin{array}{llll} W_{h} & :=\{\omega_{h}\in H_{0,D}(\text{div},\Omega ):{\left(\omega_{h}\right)}_{|T}\in {\mathbb{P}}^{0}(T,{\mathbb{R}}^{3})+x{\mathbb{P}}^{0}(T,\mathbb{R})\;\forall T\in {𝒯}_{h}\},\; & & \end{array}$$

providing the missing coupling degree of freedom per facet. Motivated by the fact that the divergence of $\omega_{h}=\text{curl}(u)\in H(\text{div},\Omega )$ vanishes for the true solution $u\in H^{1}(\Omega ,{\mathbb{R}}^{d})$, a consistent stabilizing term ${\left(\text{div}(\omega_{h}),\text{div}(\eta_{h})\right)}_{0}$ was added to the bilinear form. We only briefly sketch how to adapt the preconditioners and their analysis developed here. Since $W_{h}\subseteq H(\text{div},\Omega )$ has a coupling degree of freedom per facet, $\omega_{h}$ remains after static condensation and A is a system for $(u_{h},{û}_{h},\omega_{h})\in {𝒱}_{h}^{\text{lo}}:=V_{h}\times {\hat{V}}_{h}\times W_{h}$. The norm in ${𝒱}_{h}^{\text{lo}}$ is

$$\begin{array}{llll} \|(u_{h},{û}_{h},\omega_{h}){\|}_{\varepsilon ,h,\text{lo}}^{2}:=\underset{T\in {𝒯}_{h}}{\sum }(\|\varepsilon (u_{h}){\|}_{T}^{2}+\underset{F\in {ℱ}_{T}}{\sum } & h^{-1}\|\Pi_{F}^{0}{\left(u_{h}-û\right)}_{h}{\|}_{F}^{2}+h\|{\left(\text{curl}(u_{h})-\omega_{h}\right)}_{n}{\|}_{F}^{2}),\; & & \end{array}$$

this is justified by the discrete Korn inequality

$$\begin{array}{ll} \underset{T\in {𝒯}_{h}}{\sum }\|\nabla u_{h}{\|}_{T}^{2}≲\underset{T\in {𝒯}_{h}}{\sum }\|\varepsilon (u_{h}){\|}_{T}^{2}+\underset{F\in {ℱ}_{h}}{\sum }h^{-1}\|\Pi_{F}^{0}{⟦u_{h}⟧}_{t}{\|}_{F}^{2}+h\|⟦n\cdot \text{curl}(u_{h})⟧{\|}_{F}^{2}, & \end{array}$$

introduced in Reference 9 [lemma 3.1]. We only need to change the "embedding" operator E which now has a $W_{h}$ component and projects into the ${\hat{V}}_{h}$ component as for $u_{h}\in {\bar{V}}_{h}$ the piecewise ${\mathbb{P}}^{1}$ tangential trace ${\left({ū}_{h}\right)}_{t}\notin {\hat{V}}_{h}$,

$$\begin{array}{ll} E:{\bar{V}}_{h}\to {𝒱}_{h}^{\text{lo}}:{ū}_{h}\mapsto ({ū}_{h},\Pi_{F}^{0}{\left({ū}_{h}\right)}_{t},\text{curl}({ū}_{h})). & \end{array}$$

The analysis also needs to be only slightly modified using the equivalence

$$\begin{array}{llll} \underset{T\in {𝒯}_{h}}{\sum }\|\varepsilon (u_{h}){\|}_{T}^{2} & +\underset{F\in {ℱ}_{h}}{\sum }h^{-1}\|\Pi_{F}^{R}{⟦u_{h}⟧}_{t}{\|}_{F}^{2}∼\underset{T\in {𝒯}_{h}}{\sum }\|\varepsilon (u_{h}){\|}_{T}^{2}+\underset{F\in {ℱ}_{h}}{\sum }h^{-1}\|\Pi_{F}^{0}{⟦u_{h}⟧}_{t}{\|}_{F}^{2}+h\|\Pi_{F}^{0}⟦n\cdot \text{curl}(u_{h})⟧{\|}_{F}^{2},\; & & \end{array}$$

introduced together with the Korn inequality in Reference 9 [lemma 3.1].

## 8 NUMERICAL RESULTS

We now present numerical results that were achieved using the Netgen/NGSolve meshing and Finite Element software,^34^, ^35^ and the Algebraic Multigrid extension library NgsAMG,^36^ available from References 37, 38. The computations were performed on the Vienna Scientific Cluster (VSC4).

We considered two problems, the first of which is a standard benchmark problem from the literature (see Reference 39) where we investigate the relative performance of different ASP variations and demonstrate robustness in the polynomial degree. The second problem is a flow around an airplane model and is meant to demonstrate the effectiveness of the method even in less academic situations. For both cases, the viscosity is fixed to $\nu =10^{-3}$, the preconditioner in the conforming auxiliary space ${\hat{V}}_{h}$ was given by a single Algebraic Multigrid V‐cycle. Instead of the difficult to parallelize Block‐Gauss‐Seidel smoothers in ${\hat{A}}_{m}$ and ${\hat{A}}_{m}^{\partial }$, we use block versions of the scalable semi‐multiplicative $\ell_{1}$‐smoothers from Reference 40.

**FIGURE 1:**
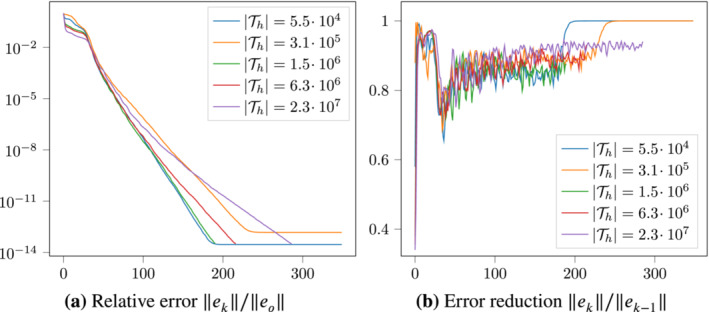
Relative errors (left) and error reduction (right) on the y‐axis versus iteration number on the x‐axis for the setup from Section 8.1.1 on the condensed system, as in Table 1 on the right. Only every other computation is shown.

Although ${\hat{K}}^{-1}$ is symmetric (see Equation 40) and would be suitable for MINRES, we use GMRES as the Krylov space method since in our experience it performs better, perhaps due to the explicit orthogonalization in every iteration. We chose to solve with a relative tolerance of $10^{-6}$ as is used commonly in the AMG literature (e.g., References 41, 42); Figure 1 demonstrates that we generally do not suffer notable loss of convergence until the error has been reduced by more than a factor of $10^{-12}$.

We show weak scaling results and therefore aim to keep the number of elements per core constant, however are only able to ensure this approximately because of the unstructured simplicial meshes we use.

**TABLE 1:** Comparison of multiplicative ASPs for the full system A and the condensed system $A^{\partial }$ for the channel problem with k=2.

			Full system				Condensed system			
$\left|{𝒯}_{h}\right|$	#D	#P	#IT	$t_{\text{tot}}$	$t_{\sup}$	$t_{\text{sol}}$	#IT	$t_{\text{tot}}$	$t_{\sup}$	$t_{\text{sol}}$
5.5$\cdot 10^{4}$	1.9$\cdot 10^{6}$	7	166	67.2	9.0	58.2	76	29.3	9.5	19.8
1.8$\cdot 10^{5}$	6.3$\cdot 10^{6}$	19	119	58.2	10.2	48.0	63	32.4	11.3	21.1
3.1$\cdot 10^{5}$	1.1$\cdot 10^{7}$	36	235	163.1	9.1	154.0	92	59.1	10.2	48.9
7.9$\cdot 10^{5}$	2.7$\cdot 10^{7}$	81	128	110.6	11.8	98.8	65	53.0	12.3	40.7
1.5$\cdot 10^{6}$	5.0$\cdot 10^{7}$	166	159	134.9	10.6	124.3	73	54.5	10.8	43.7
3.5$\cdot 10^{6}$	1.2$\cdot 10^{8}$	408	172	171.1	11.3	159.8	78	65.3	11.8	53.5
6.3$\cdot 10^{6}$	2.2$\cdot 10^{8}$	720	164	141.7	11.1	130.6	74	55.2	11.9	43.3
1.2$\cdot 10^{7}$	4.0$\cdot 10^{8}$	1333	164	169.2	12.0	157.2	75	68.0	12.9	55.1
2.3$\cdot 10^{7}$	7.9$\cdot 10^{8}$	2667	193	430.2	24.0	406.2	81	78.6	14.8	63.8

The obtained results, listed in Tables 1, 2, 3 will be discussed in detail below. For every computation we list the number of elements in the mesh $\left|{𝒯}_{h}\right|$ and the number of cores #P. With the $\sum_{h}$ dofs condensed out of the system, the relevant number of dofs is that of ${𝒱}_{h}\times Q_{h}$ which we list as #D. We give the number of iterations of GMRES needed as #IT and the total time to solve the problem (excluding loading of the mesh) as $t_{\text{tot}}$. The latter is further subdivided into the setup time $t_{\sup}$, which includes the assembly of all finite element matrices and the setup of the preconditioner, and the time $t_{\text{sol}}$ taken for GMRES iterations.

**TABLE 2:** Comparison of additive and multiplicative ASPs for $A^{\partial }$ for the channel problem with k=2.

			Additive				Multiplicative			
$\left|{𝒯}_{h}\right|$	#D	#P	#IT	$t_{\text{tot}}$	$t_{\sup}$	$t_{\text{sol}}$	#IT	$t_{\text{tot}}$	$t_{\sup}$	$t_{\text{sol}}$
5.5$\cdot 10^{4}$	1.9$\cdot 10^{6}$	5	191	60.9	13.9	47.0	75	46.0	14.2	31.8
2.1$\cdot 10^{5}$	7.3$\cdot 10^{6}$	17	191	75.8	12.8	63.0	73	45.6	14.3	31.3
4.4$\cdot 10^{5}$	1.5$\cdot 10^{7}$	35	206	154.4	13.4	141.0	77	65.7	14.6	51.1
1.5$\cdot 10^{6}$	5.0$\cdot 10^{7}$	111	169	132.8	14.0	118.7	73	73.1	15.2	57.9
6.3$\cdot 10^{6}$	2.2$\cdot 10^{8}$	480	179	176.0	15.7	160.3	74	90.9	17.0	73.9
1.4$\cdot 10^{7}$	4.6$\cdot 10^{8}$	1040	209	261.5	16.7	244.8	78	120.0	18.0	101.9
3.5$\cdot 10^{7}$	1.2$\cdot 10^{9}$	2698	230	400.2	18.0	382.3	88	159.4	20.3	139.13
5.1$\cdot 10^{7}$	1.7$\cdot 10^{9}$	3876	202	301.4	18.1	283.3	84	151.9	19.4	132.5

**TABLE 3:** Multiplicative ASP for $A^{\partial }$ with two smoothing steps for the channel problem and varying polynomial order k.

		k=1					k=2				
$\left|{𝒯}_{h}\right|$	#P	#D	#IT	$t_{\text{tot}}$	$t_{\sup}$	$t_{\text{sol}}$	#D	#IT	$t_{\text{tot}}$	$t_{\sup}$	$t_{\text{sol}}$
8.6$\cdot 10^{3}$	1	1.2$\cdot 10^{5}$	86	11.6	4.2	7.4	3.1$\cdot 10^{5}$	49	23.9	7.9	16.0
3.2$\cdot 10^{5}$	36	4.2$\cdot 10^{6}$	82	21.6	5.8	15.8	1.1$\cdot 10^{7}$	53	42.8	10.2	32.6
7.6$\cdot 10^{5}$	85	1.0$\cdot 10^{7}$	79	23.7	6.6	17.2	2.6$\cdot 10^{7}$	52	54.2	12.0	42.2
2.0$\cdot 10^{6}$	225	2.7$\cdot 10^{7}$	81	27.4	6.9	20.5	6.9$\cdot 10^{7}$	57	61.2	12.1	49.1
6.4$\cdot 10^{6}$	712	8.4$\cdot 10^{7}$	82	30.4	7.3	23.2	2.2$\cdot 10^{8}$	58	67.9	12.8	55.2
2.1$\cdot 10^{7}$	2347	2.8$\cdot 10^{8}$	85	32.6	8.2	24.3	7.2$\cdot 10^{8}$	62	74.8	14.7	60.1
4.6$\cdot 10^{7}$	5165	6.1$\cdot 10^{8}$	97	44.8	12.0	32.8	1.6$\cdot 10^{9}$	75	92.1	15.0	77.1
6.5$\cdot 10^{7}$	7168	8.4$\cdot 10^{8}$	98	43.1	10.2	32.9	2.2$\cdot 10^{9}$	75	94.6	16.1	78.5
9.7$\cdot 10^{7}$	10775	1.3$\cdot 10^{9}$	106	66.0	30.1	35.9					
		k=4									
8.6$\cdot 10^{3}$	11	1.2$\cdot 10^{6}$	63	40.8	19.4	21.4					
4.5$\cdot 10^{4}$	57	6.3$\cdot 10^{6}$	65	48.8	22.9	25.9					
1.8$\cdot 10^{5}$	227	2.5$\cdot 10^{7}$	65	63.7	28.2	35.5					
3.2$\cdot 10^{5}$	397	4.3$\cdot 10^{7}$	67	61.4	27.0	34.4					
7.6$\cdot 10^{5}$	953	1.0$\cdot 10^{8}$	65	65.7	27.4	38.3					
1.7$\cdot 10^{6}$	2064	2.2$\cdot 10^{8}$	66	61.7	24.8	36.9					
6.4$\cdot 10^{6}$	8008	8.7$\cdot 10^{8}$	64	68.7	27.7	41.0					

### 8.1 Flow around a cylinder

**FIGURE 2:**
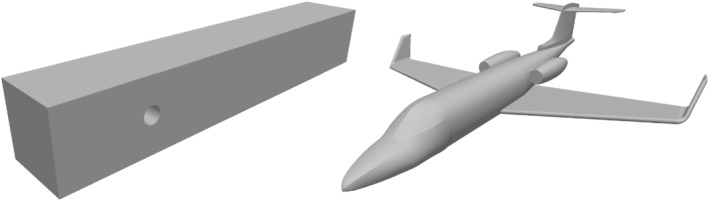
Channel with cylindrical obstacle (left) and airplane model (right).

This first series of computations concerns the flow around a cylinder as in Reference 39. The cuboid‐shaped channel Ω with cylindrical obstacle $\Omega_{c}$, $\Omega :=(0,2.5)\times (0,0.41)\times (0,0.41)\setminus {\bar{\Omega }}_{c}$ is depicted on the left in Figure 2. The boundary parts are $\Gamma_{N}=\emptyset$, $\Gamma_{Ñ}=\{(2.5,y,z)\in \partial \Omega \}$ with $\Gamma_{D}=\Gamma_{\text{in}}\cup \Gamma_{\text{wall}}$ split into inflow boundary $\Gamma_{\text{in}}:=\{(0,y,z)\in \partial \Omega \}$, where we impose a parabolic velocity inflow and wall boundary $\Gamma_{\text{wall}}$ with homogenous Dirichlet conditions.

#### 8.1.1 Full versus condensed system

We first discuss whether preconditioning A via $A^{\partial }$ as described in Section 6.1 is purely convenient for theory or also advantageous in practice. For that, we compare the multiplicative ASPs over a range of problem sizes and fixed polynomial degree k=2. As can be clearly seen in Table 1, preconditioning via the condensed system leads to considerably better performance and is the approach we take from here on out.

#### 8.1.2 Additive versus multiplicative ASP

The second choice is between additive and multiplicative ASPs, we again fix the polynomial degree to k=2 for the comparison in Table 2. From the results it is once again clear that the multiplicative preconditioner is superior and our method of choice going forward.

#### 8.1.3 High order robustness

Finally, we demonstrate robustness in the polynomial degree k with results for k∈{1,2,4}.

For the lowest order case k=1, we cannot use the MCS method (23) from Section 4 since it is simply not stable, see also Remark 1. Instead, we employ the minimal order MCS method from Reference 9, see Section 7, where, analogous to (23), we added the consistent term $\nu d^{-1}(\text{div}(u_{h}),\text{div}(v_{h}))$.

Our choice of preconditioner, informed by previous results, is the multiplicative ASP for the condensed system, this time with two smoothing steps. Due to considerably increased memory requirements, different meshes were used for k=4 than for k=1,2.

### 8.2 Flow around an airplane model

The computational domain Ω here is the “air” in a cuboid‐shaped box surrounding an airplane model $\Omega_{p}$ depicted in Figure 2, we have $\Omega =(-8,10)\times (-7,7)\times (-3,4)\setminus \Omega_{p}$. The airplane itself is contained in the bounding box [−5.2,5.3]×[−4.9,4,9]×[−0.5,1.6]. Boundary conditions, similar to the last case, are imposed velocity inflow conditions on the side of the box in front of the plane $\Gamma_{\text{in}}$, and homogenous Dirichlet conditions on $\partial \Omega_{p}$. The rest of the boundary is considered as outflow, that is, $\Gamma_{Ñ}=\partial \Omega \setminus (\Gamma_{\text{in}}\cup \partial \Omega_{p})$. The results can be found in Table 4.

**TABLE 4:** Results for the model airplane problem, k=2, multiplicative ASP for $A^{\partial }$, two smoothing steps.

$\left|{𝒯}_{h}\right|$	#D	#P	#IT	$t_{\text{tot}}$	$t_{\sup}$	$t_{\text{sol}}$
9.2$\cdot 10^{5}$	3.2$\cdot 10^{7}$	63	61	68.4	24.2	44.3
1.4$\cdot 10^{6}$	5.0$\cdot 10^{7}$	99	41	55.8	25.1	30.7
1.9$\cdot 10^{6}$	6.7$\cdot 10^{7}$	132	44	67.4	26.5	40.8
3.7$\cdot 10^{6}$	1.3$\cdot 10^{8}$	253	41	65.7	27.4	38.3
1.2$\cdot 10^{7}$	4.0$\cdot 10^{8}$	876	50	72.3	25.1	47.2
1.6$\cdot 10^{7}$	5.3$\cdot 10^{8}$	1176	52	79.1	25.6	53.5
3.0$\cdot 10^{7}$	1.0$\cdot 10^{9}$	2261	53	82.6	26.6	56.0

## 9 CONCLUSIONS

In this work, we introduced and analyzed a series of auxiliary space preconditioners for certain mass conserving stress‐yielding discretizations of the Stokes equations. In the norm induced by these MCS methods, the analysis is mostly explicit in the polynomial degree and even yields completely explicit results in the norm induced by certain hybrid discontinuous Galerkin methods that feature optimal stabilization. Numerical experiments demonstrate the robustness of the preconditioners in the polynomial degree.

## CONFLICT OF INTEREST STATEMENT

The authors declare that they have no conflicts of interest.

## Figures and Tables

**FIGURE A1:**
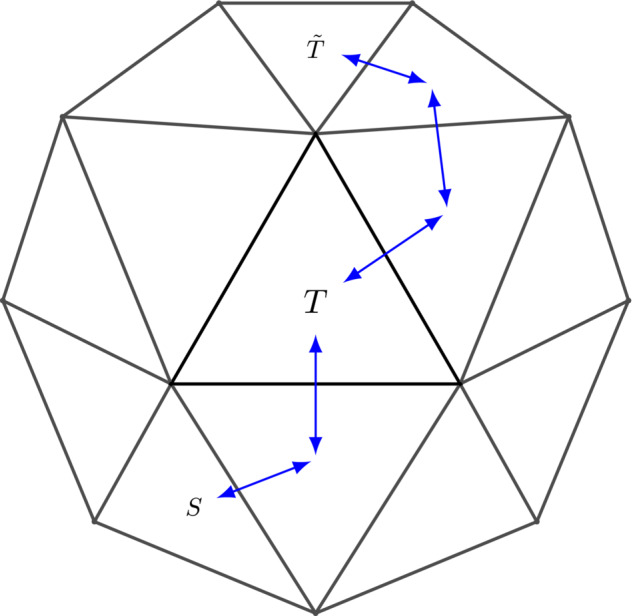
Example path in two dimensions for the last estimate in (A4).

## Data Availability

The data that support the findings of this study are available from the corresponding author upon reasonable request.

## APPENDIX A INTERPOLATION

### A.1

A standard result for ${ℐ}_{f}$, which, for example, follows from the Bramble Hilbert Lemma, the bounds for jump terms arising from nodal averaging for ${\mathbb{P}}^{1}({𝒯}_{h},{\mathbb{R}}^{d})$ functions in Reference 21 [section 3], and a trace inequality is

(A1)
$$\begin{array}{ll} \underset{T\in {𝒯}_{h}}{\sum }h^{-2}\|u- & {ℐ}_{f}u{\|}_{T}^{2}+\|\nabla (u-{ℐ}_{f}u){\|}_{T}^{2}\\ & ≲\underset{T\in {𝒯}_{h}}{\sum }\|\nabla u{\|}_{T}^{2}+\underset{F\in {ℱ}_{h}^{0}}{\sum }h^{-1}\|\Pi^{0}⟦u⟧{\|}_{F}^{2} \end{array}\;\forall u\in H^{2}({𝒯}_{h},{\mathbb{R}}^{d}).$$

Following Reference 21 [section 3], for $T\in {𝒯}_{h}$ we define $E_{T}:H^{1}(T,{\mathbb{R}}^{d})\to \text{RM}(T)$ by

$$\begin{array}{llll} \int_{T}(u-E_{T}u)\cdot q & =0\;\forall q\in {\mathbb{P}}^{0}(T,{\mathbb{R}}^{d})\;\text{and}\;\int_{T}(\text{curl}\;u-\text{curl}(E_{T}u))\cdot q=0\;\forall q\in {\mathbb{P}}^{0}(T,{\mathbb{R}}^{d(d-1)/2}),\; & & \end{array}$$

that is $\text{curl}(E_{T}u)=\Pi_{T}^{0}\text{curl}\;u$ and $\Pi_{T}^{0}E_{T}u=\Pi_{T}^{0}u$, such that (also Reference 21 [section 3])

(A2)
$$\begin{array}{ll} h^{-2}{\left\|u-E_{T}u\right\|}_{T}^{2}+{\left\|\nabla (u-E_{T}u)\right\|}_{T}^{2} & ≲{\left\|\varepsilon (u)\right\|}_{T}^{2}. \end{array}$$

With the element center of mass $x_{T}:=\Pi_{T}^{0}x$, elementary calculations show

$$\begin{array}{ll} E_{T}u(x)=\Pi_{T}^{0}u+\kappa (\Pi_{T}^{0}\text{curl}\;u)\cdot (x-x_{T}). & \end{array}$$

Proof of Lemma 1 For any $T\in {𝒯}_{h}$, define the set of element patch elements ${𝒯}_{h,T}:=\{S\in {𝒯}_{h}:\bar{T}\cap \bar{S}\neq \emptyset \}$ and the element patch $\omega_{T}:={{(⋃}_{S\in {𝒯}_{h,T}}\bar{S})}^{\circ }$. We write ${ℐ}_{\omega }$ for the local interpolation operator defined only on ${𝒯}_{h,T}$ as ${ℐ}_{f}$ is on ${𝒯}_{h}$, that is, by averaging only over values from elements in ${𝒯}_{h,T}$. There holds ${\left({ℐ}_{f}u\right)}_{|T}={\left({ℐ}_{\omega }u\right)}_{|T}$ and

$$\begin{array}{ll} {\left((I-{ℐ}_{f})u\right)}_{|T}={\left((I-{ℐ}_{\omega })(u-R)\right)}_{|T}\;\forall R\in {\mathbb{P}}^{1}(\omega_{T},{\mathbb{R}}^{d})⊇\text{RM}(\omega_{T}). & \end{array}$$

In combination with estimate (A1) applied to ${ℐ}_{\omega }$ on ${𝒯}_{h,T}$ this shows

$$\begin{array}{llll} h^{-2}\|(I-{ℐ}_{f})u{\|}_{T}^{2}+\|\nabla ((I-{ℐ}_{f})u){\|}_{T}^{2} & \leq \underset{R\in \text{RM}(\omega_{T})}{\inf}h^{-2}\|(I-{ℐ}_{\omega })(u-R){\|}_{\omega_{T}}^{2}+\|\nabla ((I-{ℐ}_{\omega })(u-R)){\|}_{\omega_{T}}^{2}\; & & \\ & ≲\underset{R\in \text{RM}(\omega_{T})}{\inf}\underset{\tilde{T}\in {𝒯}_{h,T}}{\sum }\|\nabla (u-R){\|}_{\tilde{T}}^{2}+\underset{F\in {ℱ}_{\omega }^{\circ }}{\sum }h^{-1}\|\Pi^{0}⟦u-R⟧{\|}_{F}^{2}\; & & \\ & =\underset{R\in \text{RM}(\omega_{T})}{\inf}\underset{\tilde{T}\in {𝒯}_{h,T}}{\sum }\|\nabla (u-R){\|}_{\tilde{T}}^{2}+\underset{F\in {ℱ}_{\omega }^{\circ }}{\sum }h^{-1}\|\Pi^{0}⟦u⟧{\|}_{F}^{2},\; & & \end{array}$$

where ${ℱ}_{\omega }^{\circ }$ denotes the set of interior facets of ${𝒯}_{h,T}$. We can further bound the volume terms by inserting $\pm E_{\tilde{T}}u$ and using (A2),

$$\begin{array}{llll} \underset{\tilde{T}\in {𝒯}_{h,T}}{\sum }\|\nabla (u-R){\|}_{\tilde{T}}^{2} & ≲\underset{\tilde{T}\in {𝒯}_{h,T}}{\sum }\|\nabla (u-E_{\tilde{T}}u){\|}_{\tilde{T}}^{2}+\|\nabla (E_{\tilde{T}}u-R){\|}_{\tilde{T}}^{2}≲\underset{\tilde{T}\in {𝒯}_{h,T}}{\sum }\|\varepsilon (u){\|}_{\tilde{T}}^{2}+\|\nabla (E_{\tilde{T}}u-R){\|}_{\tilde{T}}^{2}.\; & & \end{array}$$

We see that it remains to find $R\in \text{RM}(\omega_{T})$ such that

(A3)
$$\begin{array}{ll} \underset{\tilde{T}\in {𝒯}_{h,T}}{\sum }\|\nabla (E_{\tilde{T}}u-R){\|}_{\tilde{T}}^{2}≲\underset{\tilde{T}\in {𝒯}_{h,T}}{\sum }\|\varepsilon (u){\|}_{\tilde{T}}^{2}+\underset{F\in {ℱ}_{\omega }^{\circ }}{\sum }h^{-1}\|\Pi^{R}⟦u⟧{\|}_{F}^{2}. & \end{array}$$

Similar to the definition of $E_{T}$, with $x_{\omega }:=\Pi_{\omega_{T}}^{0}x$ a suitable R is

$$\begin{array}{llll} R & :=\Pi_{\omega_{T}}^{0}u+\kappa (\Pi_{\omega_{T}}^{0}\text{curl}\;u)\cdot (x-x_{\omega }).\; & & \end{array}$$

Calculations show $\Pi_{\omega_{T}}^{0}\text{curl}\;u=\sum_{\tilde{T}\in {𝒯}_{h,T}}\alpha_{\tilde{T}}\Pi_{\tilde{T}}^{0}\text{curl}\;u$ with $\alpha_{\tilde{T}}:=\frac{\left|\tilde{T}\right|}{\left|\omega_{T}\right|}$, and therefore

$$\begin{array}{llll} R & =\Pi_{\omega_{T}}^{0}u+\underset{\tilde{T}\in {𝒯}_{h,T}}{\sum }\alpha_{\tilde{T}}\kappa (\text{curl}(E_{\tilde{T}}u))\cdot (x-x_{\omega }).\; & & \end{array}$$

As $\varepsilon (E_{\tilde{T}}u-R)=0$, there holds $\nabla (E_{\tilde{T}}u-R)=\kappa (\text{curl}(E_{\tilde{T}}u-R))\in {\mathbb{P}}^{0}({𝒯}_{h,T},{\mathbb{R}}^{d})$, i.e.

$$\begin{array}{ll} \|\nabla (E_{\tilde{T}}u-R){\|}_{\tilde{T}}^{2}∼h^{d}|\nabla {\left(E_{\tilde{T}}u-R\right)}_{|\tilde{T}}{|}^{2}=h^{d}|\underset{S\in {𝒯}_{h,T}}{\sum }\alpha_{S}(\text{curl}(E_{\tilde{T}}u)-\text{curl}(E_{S}u))|, & \end{array}$$

where we used $\sum_{S\in {𝒯}_{h,T}}\alpha_{S}=1$ and therefore, with $\alpha_{S}\leq 1$,

$$\begin{array}{llll} \underset{\tilde{T}\in {𝒯}_{h,T}}{\sum }\|\nabla (E_{\tilde{T}}u-R){\|}_{\tilde{T}}^{2} & ≲\underset{\tilde{T}\in {𝒯}_{h,T}}{\sum }\underset{S\in {𝒯}_{h,T}}{\sum }h^{d}|(\text{curl}(E_{\tilde{T}}u)-\text{curl}(E_{S}u))|.\; & & \end{array}$$

Any two elements in ${𝒯}_{h,T}$ are connected via a path over a bounded number of other elements in ${𝒯}_{h,T}$, and we can bound this last sum by one over facet terms (see Figure A1):

(A4)
$$\begin{array}{ll} \underset{\tilde{T}\in {𝒯}_{h,T}}{\sum }\underset{S\in {𝒯}_{h,T}}{\sum }h^{d}|( & \text{curl}(E_{\tilde{T}}u)-\text{curl}(E_{S}u))|≲\underset{F\in {ℱ}_{\omega }^{\circ }}{\sum }h^{d}|\text{curl}(E_{T_{F,L}}u)-\text{curl}(E_{T_{F,R}}u){|}^{2}, \end{array}$$

where $T_{F,L}$ and $T_{F,R}$ denote the two elements that share the facet F. Any facet is only summed up over a bounded number of times after reordering of the sum and due to the shape regularity of ${𝒯}_{h}$, (A4) holds with a single constant for all patches in ${𝒯}_{h}$. These jump terms can be bounded by an expansion of $E_{\tilde{T}}u$ at $x_{F}:=\Pi_{F}^{0}(x)$,

$$\begin{array}{ll} E_{\tilde{T}}u(x)=\Pi_{F}^{0}(E_{\tilde{T}}u)+\kappa (\text{curl}(E_{\tilde{T}}u))\cdot (x-x_{F})\;\text{for}\;x\in F, & \end{array}$$

and the elementary estimate $\|x-x_{F}{\|}_{F}^{2}∼h^{2}|F|∼h^{d+1}$. Writing ${𝒯}_{F}:=\{T_{F,L},T_{F,R}\}$ we see

$$\begin{array}{llll} h^{d}|\text{curl}(E_{T_{F,L}}u)-\text{curl}(E_{T_{F,R}}u){|}^{2} & ≲h^{-1}\|\kappa (\text{curl}(E_{T_{F,L}}u)-\text{curl}(E_{T_{F,R}}u))\cdot (x-x_{F}){\|}_{F}^{2}\; & & \\ & =h^{-1}\|(\Pi_{F}^{R}-\Pi_{F}^{0})(E_{T_{F,L}}u-E_{T_{F,R}}u){\|}_{F}^{2}\; & & \\ & ≲h^{-1}\|\Pi_{F}^{R}⟦u⟧{\|}_{F}^{2}+\underset{\tilde{T}\in {𝒯}_{F}}{\sum }h^{-1}\|\Pi_{F}^{R}(u-E_{\tilde{T}}u){\|}_{F}^{2}.\; & & \end{array}$$

Finally, an $H^{1}$ trace inequality and (A2) let us bound

$$\begin{array}{ll} h^{-1}\|\Pi_{F}^{R}(u-E_{\tilde{T}}u){\|}_{F}^{2}≲\|(u-E_{\tilde{T}}u){\|}_{H^{1}(\tilde{T})}^{2}≲\|\varepsilon (u){\|}_{\tilde{T}}^{2}, & \end{array}$$

in summary,

$$\begin{array}{llll} \underset{\tilde{T}\in {𝒯}_{h,T}}{\sum }\|\nabla (E_{\tilde{T}}u-R){\|}_{\tilde{T}}^{2} & ≲\underset{F\in {ℱ}_{\omega }^{\circ }}{\sum }h^{d}|\text{curl}(E_{T_{F,L}}u)-\text{curl}(E_{T_{F,R}}u){|}^{2}≲\underset{\tilde{T}\in {𝒯}_{h,T}}{\sum }\|\varepsilon (u){\|}_{\tilde{T}}^{2}+\underset{F\in {ℱ}_{\omega }^{\circ }}{\sum }h^{-1}\|\Pi_{F}^{R}⟦u⟧{\|}_{F}^{2},\; & & \end{array}$$

that is, (A3) holds for our specific choice of R which finishes the proof.

Proof of Lemma 2 An inverse estimate for the piecewise linear ${ℐ}_{f}u\in {\bar{V}}_{h}^{f}$ shows

$$\begin{array}{llll} \underset{T\in {𝒯}_{h}}{\sum }h^{-2}\|u-ℐu{\|}_{T}^{2} & ≲\underset{T\in {𝒯}_{h}}{\sum }h^{-2}\|u-{ℐ}_{f}u{\|}_{T}^{2}+h^{-2}\|(I-\pi_{0}){ℐ}_{f}u{\|}_{T}^{2}≲\underset{T\in {𝒯}_{h}}{\sum }(h^{-2}\|u-{ℐ}_{f}u{\|}_{T}^{2}+\underset{F\in {ℱ}_{T}\cap {ℱ}_{h}^{D}}{\sum }h^{-1}\|(I-\pi_{0}){ℐ}_{f}u{\|}_{F}^{2}).\; & & \end{array}$$

As $(I-\pi_{0}){ℐ}_{f}u={ℐ}_{f}u$ on $F\in {ℱ}_{h}^{D}$, and ${ℐ}_{f}u\in {\mathbb{P}}^{1}({𝒯}_{h},{\mathbb{R}}^{d})$

$$\begin{array}{llll} \|(I-\pi_{0}){ℐ}_{f}u{\|}_{F}^{2}=\|\Pi_{F}^{1}{ℐ}_{f}u{\|}_{F}^{2} & ≲\|\Pi_{F}^{R}{ℐ}_{f}u{\|}_{F}^{2}+\|(\Pi_{F}^{1}-\Pi_{F}^{R}){ℐ}_{f}u{\|}_{F}^{2}≲\|\Pi_{F}^{R}u{\|}_{F}^{2}+\|\Pi_{F}^{R}(u-{ℐ}_{f}u){\|}_{F}^{2}+\|(\Pi_{F}^{1}-\Pi_{F}^{R}){ℐ}_{f}u{\|}_{F}^{2}\; & & \end{array}$$

We bound the second one with an $H^{1}$ trace inequality and a scaling argument,

$$\begin{array}{ll} h^{-1}\|\Pi_{F}^{R}(u-{ℐ}_{f}u){\|}_{F}^{2}≲h^{-2}\|u-{ℐ}_{f}u{\|}_{T}^{2}+\|\nabla (u-{ℐ}_{f}u){\|}_{T}^{2}, & \end{array}$$

where T is the unique element such that $F\in {ℱ}_{T}$. An explicit expansion of the piecewise linear ${ℐ}_{f}u$ at the facet center of mass $x_{F}:=\Pi_{F}^{0}x$ shows

$$\begin{array}{llll} h^{-1}\|(\Pi_{F}^{1}-\Pi_{F}^{R}){ℐ}_{f}u{\|}_{F}^{2} & =h^{-1}\|\varepsilon ({\left({ℐ}_{f}u\right)}_{|T})\cdot (x-x_{F}){\|}_{F}^{2}≲\|\varepsilon ({ℐ}_{f}u){\|}_{T}^{2}≲\|\varepsilon (u){\|}_{T}^{2}+\|\nabla (u-{ℐ}_{f}u){\|}_{T}^{2}\; & & \end{array}$$

Finally, with $⟦\Pi_{F}^{R}u⟧=\Pi_{F}^{R}u$ on $F\in {ℱ}_{h}^{D}$, by Lemma 1 there holds

$$\begin{array}{llll} \underset{T\in {𝒯}_{h}}{\sum }h^{-2}\|u-ℐu{\|}_{T}^{2} & ≲\underset{T\in {𝒯}_{h}}{\sum }(h^{-2}\|u-{ℐ}_{f}u{\|}_{T}^{2}+\|\nabla (u-{ℐ}_{f}u){\|}_{T}^{2}+\|\varepsilon (u){\|}_{T}^{2}+\underset{F\in {ℱ}_{T}\cap {ℱ}_{h}^{D}}{\sum }h^{-1}\|\Pi_{F}^{R}u{\|}_{F}^{2})\; & & \\ & ≲\underset{T\in {𝒯}_{h}}{\sum }(\|\varepsilon (u){\|}_{T}^{2}+\underset{F\in {ℱ}_{T}}{\sum }h^{-1}\|\Pi_{F}^{R}⟦u⟧{\|}_{F}^{2}).\; & & \end{array}$$

The other volume terms $\|\nabla (u-ℐu){\|}_{T}$ in (17) are bounded analogously.

## APPENDIX B TRACE ESTIMATES

### B.1

The crucial step in the proof of (18) in Reference 20 was to construct for a given $w\in {\mathbb{P}}^{k}(T,\mathbb{R})$ a $\tilde{w}\in {\mathbb{P}}^{k}(T,\mathbb{R})$ that approximates it in $\|\cdot {\|}_{j,F}$ and is bounded in the $H^{1}$ semi‐norm and yet ${\tilde{w}}_{|\partial F}=0$. That is, implicitly, for $F\in {ℱ}_{h}$ and $T\in {𝒯}_{h}$ with $F\in {ℱ}_{T}$, an operator

(B1)
$$\begin{array}{ll} & {ℰ}_{0}^{s,F}:{\mathbb{P}}^{k}(T,\mathbb{R})\to {\mathbb{P}}^{k}(T,\mathbb{R})\;\text{with}\;{\left({ℰ}_{0}^{s,F}u\right)}_{|\partial F}=0\\ & \text{and}\;\|\nabla {ℰ}_{0}^{s,F}u{\|}_{T}^{2}+\|{ℰ}_{0}^{s,F}u-u{\|}_{j,F}^{2}≲\log k\|u{\|}_{H^{1}(T)}^{2} \end{array}$$

was constructed. As no boundary conditions are enforced strongly in $\|\cdot {\|}_{1,F,0}$, the trace of ${ℰ}_{0}^{s,F}w$ could be extended to a function admissible for the infimum in $\|\cdot {\|}_{1,F,0}$. In addition to ${ℰ}_{0}^{s,F}$, of the commuting $H^{1},H(\text{curl})$ and H(div) extensions introduced in References 43, 44, 45, we need the first,

$$\begin{array}{llll} & {ℰ}^{s}:{\mathbb{P}}^{k}(\partial T,\mathbb{R})\to {\mathbb{P}}^{k}(T,\mathbb{R})\; & & \\ & \text{with}\;{\left({ℰ}^{s}u\right)}_{\partial T}=u_{\partial T},\;\text{and}\;\|{ℰ}^{s}u{\|}_{H^{1}(T)}^{2}≲\|u{\|}_{H^{1/2}(\partial T)}^{2},\; & & \end{array}$$

see Reference 43 [theorem 6.1], and last,

$$\begin{array}{llll} & {ℰ}^{\text{div}}:\{u\in H^{-1/2}(\partial T):u_{|F}\in {\mathbb{P}}^{k}(F,{\mathbb{R}}^{d})\;\forall F\in {ℱ}_{T}\}\to {\mathbb{P}}^{k}(T,{\mathbb{R}}^{d})\; & & \\ & \text{with}\;{\left({\left({ℰ}^{\text{div}}u\right)}_{n}\right)}_{|F}={\left(u_{n}\right)}_{|F}\;F\in {ℱ}_{T},\;\text{and}\;\|{ℰ}^{\text{div}}u{\|}_{H^{1}(T)}^{2}≲\|u{\|}_{H^{-1/2}(F)}^{2}.\; & & \end{array}$$

Note although that ${ℰ}^{\text{div}}$ was constructed from $H^{-1/2}(\partial T)\to H^{\text{div}}(T)$ in Reference 44 [theorem 7.1], the authors actually proved continuity in the $H^{1}(T)$ norm.

Lemma 10 For $F\in {ℱ}_{h}$ and $u\in {\mathbb{P}}^{k}(T,{\mathbb{R}}^{d})$ there exists $ũ\in {\mathbb{P}}^{k}(T,{\mathbb{R}}^{d})$ with ${ũ}_{n}=u_{n}$ on F and ${ũ}_{n}=0$ on $\tilde{F}\in {ℱ}_{T}\setminus \{F\}$ such that

(B2)
$$\begin{array}{ll} \|\nabla ũ{\|}_{T}^{2}+\|{\left(ũ-u\right)}_{t}{\|}_{j,F}^{2}+\underset{\tilde{F}\in {ℱ}_{T}\setminus \{F\}}{\sum }h^{-1}\|u_{t}{\|}_{j,\tilde{F}}^{2}≲{\left(\log k\right)}^{3}\|u{\|}_{H^{1}(T)}^{2}. & \end{array}$$

To simplify the notation we only show a proof in three dimensions, the two‐dimensional case works analogously. It also suffices to show the estimate on a reference tetrahedron, for general tetrahedra it follows from scaling arguments. First, we use ${ℰ}^{\text{div}}$ to get a ${ũ}_{1}$ which fulfills ${\left({ũ}_{1}\right)}_{n}=u_{n}$ on F and ${\left({ũ}_{1}\right)}_{n}=0$ on $\tilde{F}\in {ℱ}_{T}\setminus \{F\}$ with a bounded $H^{1}$ norm,

$$\begin{array}{ll} \|{ũ}_{1}{\|}_{H^{1}(T)}^{2}≲\|u{\|}_{H^{-1/2}(F)}^{2}≲\|u{\|}_{H(\text{div},T)}^{2}\leq \|u{\|}_{H^{1}(T)}^{2}. & \end{array}$$

We have no control over the tangential traces of ${ũ}_{1}$ and have to add correction terms. For all $\hat{F}\in {ℱ}_{T}$ we pick two arbitrary normalized, orthogonal tangent vectors $t_{\hat{F}}$ and ${\tilde{t}}_{\hat{F}}$ and write the “errors” we need to compensate for on F and $\tilde{F}\in {ℱ}_{T}\setminus \{F\}$ as

$$\begin{array}{llll} & \lambda_{F}t_{F}:=((u-{ũ}_{1})\cdot t_{F})t_{F},\;\;{\tilde{\lambda }}_{F}{\tilde{t}}_{F}:=((u-{ũ}_{1})\cdot {\tilde{t}}_{F}){\tilde{t}}_{F},\; & & \\ & \lambda_{\tilde{F}}t_{\tilde{F}}:=-({ũ}_{1}\cdot t_{\tilde{F}})t_{\tilde{F}},\;\;{\tilde{\lambda }}_{\tilde{F}}{\tilde{t}}_{\tilde{F}}:=-({ũ}_{1}\cdot {\tilde{t}}_{\tilde{F}}){\tilde{t}}_{\tilde{F}}.\; & & \end{array}$$

We write ${ℰ}^{s,\hat{F}}$ for ${ℰ}^{s}$ applied to the extension by zero to ∂T of functions that vanish on $\partial \hat{F}$. This defines an $H_{00}^{1/2}$ stable extension

$$\begin{array}{ll} {ℰ}^{s,\hat{F}}:{\mathbb{P}}^{k}(F,\mathbb{R})\cap H_{00}^{1/2}(F)\to {\mathbb{P}}^{k}(T,\mathbb{R})\;\text{with}\;\|{ℰ}^{s,\hat{F}}u{\|}_{H^{1}(T)}≲\|u{\|}_{H_{00}^{1/2}(F)}, & \end{array}$$

and construct a corrected ũ as

$$\begin{array}{llll} ũ\;:= & {ũ}_{1}+\underset{\hat{F}\in {ℱ}_{T}}{\sum }{ℰ}^{s,\hat{F}}{ℰ}_{0}^{s,\hat{F}}(\lambda_{F})t_{\hat{F}}+{ℰ}^{s,\hat{F}}{ℰ}_{0}^{s,\hat{F}}({\tilde{\lambda }}_{F}){\tilde{t}}_{\hat{F}},\; & & \end{array}$$

where we understand ${ℰ}^{s,\hat{F}}$ to be applied to the respective trace on $\hat{F}$. The added corrections are normal bubbles because on their associated facet they are a scalar times a tangential and their trace vanishes on all others, that is, ${ũ}_{n}={\left({ũ}_{1}\right)}_{n}\;\forall F\in {ℱ}_{T}$ and ũ is admissible and we need to show that if fulfills (B2). As ${ℰ}^{s,\tilde{F}}$ restricted to $\tilde{F}$ is just the identity, for $\tilde{F}\in {ℱ}_{T}\setminus \{F\}$ there holds

$$\begin{array}{ll} \|ũ\cdot t_{\tilde{F}}{\|}_{j,\tilde{F}}^{2}=\|({ũ}_{1}\cdot t_{\tilde{F}})-{ℰ}^{s,\tilde{F}}{ℰ}_{0}^{s,\tilde{F}}({ũ}_{1}\cdot t_{\tilde{F}}){\|}_{j,\tilde{F}}^{2}=\|(I-{ℰ}_{0}^{s,\tilde{F}})({ũ}_{1}\cdot t_{\tilde{F}}){\|}_{j,\tilde{F}}^{2}, & \end{array}$$

and (B1) implies

$$\begin{array}{ll} \|ũ\cdot t_{\tilde{F}}{\|}_{j,\tilde{F}}^{2}≲\log k\|{ũ}_{1}\cdot t_{\tilde{F}}{\|}_{H^{1}(T)}^{2}≲\log k\|{ũ}_{1}{\|}_{H^{1}(T)}^{2}≲\log k\|u{\|}_{H^{1}(T)}^{2}. & \end{array}$$

The volume terms arising from the correction of $\lambda_{\tilde{F}}$ for $\tilde{F}\in {ℱ}_{T}\setminus \{F\}$ can be bounded with the inverse estimate $\|v{\|}_{H_{00}^{1/2}(\tilde{F})}≲{\left(\log k\right)}^{2}\|v{\|}_{H^{1/2}(\tilde{F})}$ for polynomials that vanish on $\partial \tilde{F}$, see Reference 45 [lemma 4.7],

$$\begin{array}{ll} \|\nabla {ℰ}^{s,\tilde{F}}{ℰ}_{0}^{s,\tilde{F}}(\lambda_{\tilde{F}})t_{\tilde{F}}{\|}_{T}^{2}≲\|{ℰ}_{0}^{s,\tilde{F}}\lambda_{\tilde{F}}{\|}_{H_{00}^{1/2}(\tilde{F})}^{2}≲{\left(\log k\right)}^{2}\|{ℰ}_{0}^{s,\tilde{F}}\lambda_{\tilde{F}}{\|}_{H^{1/2}(\tilde{F})}^{2}, & \end{array}$$

and we can continue with (B1) to see

$$\begin{array}{ll} \|\nabla {ℰ}^{s,\tilde{F}}{ℰ}_{0}^{s,\tilde{F}}(\lambda_{\tilde{F}})t_{\tilde{F}}{\|}_{T}^{2}≲{\left(\log k\right)}^{3}\|{ũ}_{1}\cdot t_{\tilde{F}}{\|}_{H^{1}(T)}^{2}≲{\left(\log k\right)}^{3}\|u{\|}_{H^{1}(T)}^{2}. & \end{array}$$

Analogously, we show these bounds for volume and trace terms for $\tilde{F}=F$ as well as ${\tilde{t}}_{\left(\cdot \right)},{\tilde{\lambda }}_{\left(\cdot \right)}$ instead of $t_{\left(\cdot \right)},\lambda_{\left(\cdot \right)}$. In summary, we have

$$\begin{array}{llll} \|\nabla ũ{\|}_{T}^{2} & +\|{\left(u-ũ\right)}_{t}|{|}_{j,F}^{2}+\underset{\tilde{F}\in {ℱ}_{T}\setminus \{F\}}{\sum }\|{ũ}_{t}{\|}_{j,\tilde{F}}^{2}\; & & \\ ≲\; & \|\nabla {ũ}_{1}{\|}_{F}^{2}+\underset{\hat{F}\in {ℱ}_{T}}{\sum }\|\nabla {ℰ}^{s,\hat{F}}{ℰ}_{0}^{s,\hat{F}}(\lambda_{\hat{F}})t_{\hat{F}}{\|}_{T}^{2}+\|\nabla {ℰ}^{s,\hat{F}}{ℰ}_{0}^{s,\hat{F}}({\tilde{\lambda }}_{\hat{F}}){\tilde{t}}_{\hat{F}}{\|}_{T}^{2}+\|{\left(u-ũ\right)}_{t}|{|}_{j,F}^{2}+\underset{\tilde{F}\in {ℱ}_{T}\setminus \{F\}}{\sum }\|{ũ}_{t}{\|}_{j,\tilde{F}}^{2}\; & & \\ ≲\; & {\left(\log k\right)}^{3}\|u{\|}_{H^{1}(T)}^{2}.\; & & \end{array}$$

Proof of Lemma 1 For the minimizer w in (19), a Korn inequality on T shows

$$\begin{array}{llll} \|w{\|}_{H^{1}(T)}^{2} & ≲\|\varepsilon (w){\|}_{T}^{2}+\|\Pi_{F}^{R}w{\|}_{j,F}^{2}=\|\varepsilon (w){\|}_{T}^{2}+\|\Pi_{F}^{R}{\left(w-û\right)}_{t}{\|}_{j,F}^{2}≲\|(u,û){\|}_{\varepsilon ,F}.\; & & \end{array}$$

Choosing $\tilde{w}\in {\mathbb{P}}^{k}(T,{\mathbb{R}}^{d})$ as in Lemma 10, finishes the proof as it is admissible for the infimum in (20) and bounds it by $\|w{\|}_{H^{1}(T)}^{2}≲\|(u,û){\|}_{\varepsilon ,F}$.
